# Plateaus in the Potentials of Density-Functional Theory: Analytical Derivation and Useful Approximations

**DOI:** 10.1021/acs.jctc.4c01771

**Published:** 2025-03-27

**Authors:** Nathan E. Rahat, Eli Kraisler

**Affiliations:** † Fritz Haber Research Center for Molecular Dynamics and Institute of Chemistry, The Hebrew University of Jerusalem, 9091401 Jerusalem, Israel

## Abstract

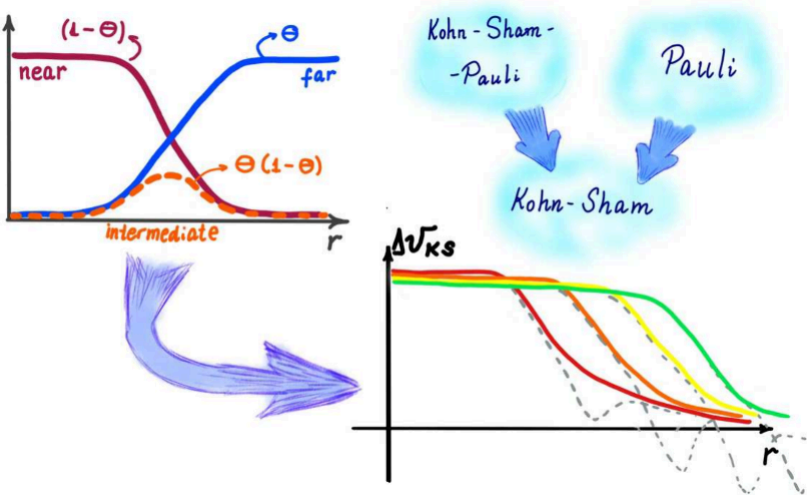

Density functional theory (DFT) is an extremely efficient and widely used method for electronic structure calculations. However, the quality of such calculations crucially depends on the quality of the approximation used for the exchange-correlation functional, for which there is no exact form. One important feature of the exact exchange-correlation potential, which common approximations usually do not capture, is the spatial steps and plateaus that occur in various scenarios, including ionization, excitation, dissociation, and charge transfer. In this paper, we derive an analytical expression for the plateau in the Kohn–Sham potential that forms around the center of the system, when the number of electrons infinitesimally surpasses an integer. The resulting formula is the first analytical expression of its kind. The derivation is performed using the orbital-free DFT framework, analyzing both the Kohn–Sham–Pauli and the Pauli potentials. Analytical results are compared to exact calculations for small atomic systems, showing close correspondence and high accuracy. Furthermore, it is shown that plateaus can be produced also when relying on approximate electron densities, even those obtained with the simplest exchange-correlation form—the local density approximation.

## 1 Introduction

Describing many-electron systems accurately is a major challenge in chemistry, solid state physics and materials science. To this end, one would have to solve the many-body Schrödinger equation, whose analytical solution is not attainable for almost any system of interest. As a result, various methods to bypass the need of directly solving this equation have been developed.^1,2^

Density-functional theory (DFT)^3−6^ is a leading theoretical framework that can address the many-electron problem accurately and efficiently. Most popular is the Kohn–Sham (KS) spin-density functional approach,^7−9^ where the original system of interacting electrons is described by an auxiliary system of noninteracting electrons, with the same electron density. Solving the noninteracting Schrödinger equations, obtaining the KS orbitals and from them–the density, allows to calculate the energy and other properties of the original system of interest.

As an alternative to the KS approach, there exists the orbital-free (OF) approach in DFT,^10−13^ where (the square root of) the density is obtained directly from a Schrödinger-like equation. Due to its lower computational cost, OF-DFT has the potential of addressing systems of millions of atoms.^14−16^ Although less widespread than its KS counterpart, the OF-DFT approach lately gained much attention.^17−27^ It is closely related to the emergent exact electron factorization (EEF) method.^28−30^

Although DFT is exact in principle, all practical calculations, in both the KS and OF methods, require the use of approximations. Within KS-DFT, the main approximation is that of the exchange and correlation (xc) functional. In OF-DFT, there is a need to approximate not only exchange and correlation, but the Pauli functional, as well.

The xc potential, *v*_xc_[*n*](**r**), is the functional derivative of the xc energy with respect to the density, , and can be expressed as the difference between the KS and the external and Hartree potentials: *v*_xc_[*n*](**r**) = *v*_KS_[*n*](**r**) – *v*_ext_(**r**) – *v*_Hartree_[*n*](**r**). Broadly speaking, *v*_xc_(**r**) is responsible for all the quantum interactions between the electrons, namely all interactions that are beyond the attraction of the electrons to the nuclei and interaction with an external field (*v*_ext_) and the classical electrostatic self-repulsion of the electron density (*v*_Hartree_). The exact form of *v*_xc_(**r**) is not known, but many of its properties are. In particular, *v*_xc_(**r**) tends to form sharp spatial steps in a variety of scenarios: electron addition, dissociation, excitation and charge transfer.^31^ In the case of an electron addition, i.e., when considering a many-electron system with a varying number of electrons, *N*, it is known that as *N* surpasses an integer value, *v*_xc_(**r**) raises a plateau around the system: *v*_xc_(**r**) experiences a uniform shift upward, and drops to zero as one moves far away from the center of the system. The height of the uniform shift equals
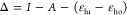
1i.e., the difference between the fundamental gap, *E*_*g*_ = *I* – *A*, and the KS gap, *E*_*g*_^KS^ = ε_lu_ – ε_ho_. The former equals the difference between the ionization potential (IP), *I*, and the electron affinity (EA), *A*, while the latter is the difference between the lowest unoccupied (lu) and the highest occupied (ho) KS energy levels. Presence of this plateau feature is essential to satisfy the IP theorem in DFT,^10,32−41^ for the exact xc functional, which is closely related to the piecewise-linearity property of the total energy as a function of the electron number, *E*(*N*). The plateau described above has been observed numerically many times (e.g.,^31,42−50^ and references therein). An early analytical study of a one-dimensional model KS potential in the form of a delta-function appears in ref (51). Furthermore, several studies^46,52,53^ analytically described steps, including in the context of molecular dissociation, in systems of *N* = 2 electrons at most, in a singlet state. In such cases, the simple relation between the density, *n*(**r**), and the only occupied KS orbital, φ_1_(**r**), namely, *n*(**r**) = *N*|φ_1_(**r**)|^2^, allowed to find the dependence of the KS potential on the density and observe the dissociation step. However, an exact, general analytic expression for the **r**- and *N*-dependence of the plateau function, for a general many-electron system, is still missing.

The Pauli potential, , is the functional derivative of the difference between the KS kinetic energy and the von Weizsäcker term, , namely between the kinetic energy of noninteracting fermions and bosons, with the same density.^4,10,12^ For this reason, *v*_P_[*n*](**r**) has long been interpreted as the term that embodies all the effects of the Pauli principle, namely conveying the information that we are treating fermions. Recently, ref (30) (Sec. II C) provided a different interpretation of *v*_P_(**r**), in the context of the EEF theory, without referring to a bosonic reference system: *v*_P_(**r**) has been identified as the difference between the EEF and the KS potentials, and can now be viewed in terms of how one electron with a given probability distribution of (*n*(**r**)/*N*)^1/2^ feels the environment provided by the neighboring electrons.

An exact, explicit expression of *v*_P_[*n*](**r**) in terms of the density, *n*(**r**), does not exist, and its accurate approximation is a central challenge in the OF-DFT approach. However, there exists an exact expression for *v*_P_(**r**) in terms of the KS orbitals and eigenvalues, originally derived by Levy and Ou-Yang in 1988^12^ and recently generalized by Kraisler and Schild^54^ for the spin-dependent case with a possibly varying number of electrons (see eq 21 below). This enabled examining the Pauli potential in the scenario of electron addition and to conclude that also *v*_P_(**r**) builds a plateau as *N* surpasses an integer. The plateau height equals the KS gap, *E*_*g*_^KS^, and it has been observed for both the exact and the approximate cases.^54^ Although the aforementioned exact expressions for *v*_P_(**r**) cannot be used directly in OF-DFT, because in that approach KS orbitals are not available, it is crucial in the derivation of the plateau function, which we attempt in this article.

A detailed description of the plateaus forming in both *v*_xc_(**r**) and *v*_P_(**r**) is an important, fundamental challenge. Understanding these properties is useful to construct advanced, more accurate approximations, preferably at moderate numerical cost. In addition, analytical knowledge about plateaus is instrumental in benchmarking numerical calculations that present plateaus and steps (see, e.g.,^31,50^), as these calculations are very sensitive in regions of low density.

In the present contribution, we analytically derive an expression for the plateau that appears in the KS potential of spin-density functional theory, in the scenario of electron addition, as *N* surpasses an integer. We do so by independently examining plateaus in two potentials: the Kohn–Sham−Pauli (KSP) potential, *v*_KSP_(**r**) (Sec. 2.1), and the Pauli potential, *v*_P_(**r**) (Sec. 2.2). The expression for the KS plateau is then obtained by subtraction. We support our derivation by numerical calculations for atomic systems. After providing the numerical details in Sec. 3, we first present the exact case, namely we obtain plateaus relying on densities obtained with full configuration-interaction (FCI) calculations (Secs. 4.1–4.4). Then, in Sec. 4.5, we discuss emergence of plateaus for approximate densities, specifically densities obtained from the simplest xc functional–the local spin density approximation (LSDA). Section 5 summarizes this work.

## 2 Theory

### 2.1 Kohn–Sham–Pauli Plateau

To derive the plateau for the KSP potential, *v*_KSP_(**r**; *N*), in the scenario of electron addition, we consider a many-electron system with a varying number of electrons, *N* = *N*_0_ + α, where *N*_0_ is an integer and α ∈ [0, 1]. We shall work within the spin-polarized DFT formalism and focus on the common case where the variation in the number of electrons happens in one of the spin channels, *N*_σ_ = *N*_σ_^0^ + α, whereas the number of electrons in the other spin channel, *N*_σ̅_ = *N*_σ̅_^0^, is kept constant (σ̅ is the spin channel opposite to σ). Transition of our findings to the spin-unpolarized case is discussed in Sec. 2.3.

We define the plateau function for the scenario of electron addition,
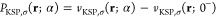
2as the difference between the KSP potentials of the σ-spin-channel for *N*_σ_ and *N*_σ_^0–^ electrons, respectively. Particularly, we are interested in the limit α → 0^+^, i.e. in comparing the KSP potentials just before and just after the number of electrons surpasses an integer value.

#### 2.1.1 Kohn–Sham–Pauli Potential

In OF-DFT, the spin-density *n*_σ_(**r**), for each spin channel, can be obtained from the following Schrödinger-like equation (see ref (10−12, 30, 54, 55), and references therein):
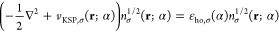
3Hartree atomic units are used throughout. We stress that the density and the KSP potential employed in eq 3 depend on the number of electrons in the system, i.e., on α. The eigenvalue on the right-hand side (rhs), ε_ho,σ_, is the highest occupied (ho) KS eigenvalue of that spin channel.

For the exact xc functional, there is a direct connection between ε_ho,σ_(α) and the ionization potential (IP) or the electron affinity (EA)^32,56,57^ (note that when we cross an integer electron number, the actual level that is named “ho” *changes*). In case *N*_0_ electrons correspond to an overall electrically neutral system (*N*_↑_^0^ and *N*_↓_^0^ electrons in each spin channel, respectively), for *N*_σ_ ∈ (*N*_σ_^0^ – 1, *N*_σ_^0^], ε_ho,σ_ equals – *I*_σ_, the σ-IP, i.e. the energy required to remove an electron with a given spin. For *N*_σ_ ∈ (*N*_σ_^0^, *N*_σ_^0^ + 1], ε_ho,σ_ equals – *A*_σ_, the σ-EA. The overall IP equals *I* = min_σ_(*I*_σ_) and the overall EA is *A* = max_σ_(*A*_σ_). For the exact case, ε_ho,σ_ does not depend on α (as long as α ∈ (0, 1]), and overall exhibits the stair-step behavior with *N*. However, for approximations ε_ho,σ_ may depend on α significantly.

By rearrangement of terms in eq 3, one can show that
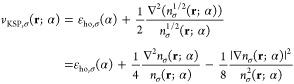
4

Next, we employ the piecewise-linearity property of the exact electron (spin-)density,^32^ which states that for any fractional value of α the density *n*_σ_(**r**; α) is a linear combination of *n*_σ_(**r**; 0) and *n*_σ_(**r**;1): This property can be expressed as
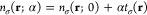
5where *t*_σ_(**r**) ≔ *n*_σ_(**r**; 1)–*n*_σ_(**r**; 0) is the Fukui function for addition of an electron (to the σ-spin-channel). Notably, *t*_σ_(**r**) is α-independent.

Substitution of eq 5 into eq 4 leads to the following expression:
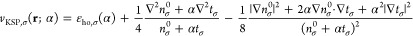
6where we denote *n*_σ_^0^ ≡ *n*_σ_(**r**; 0) and suppress the dependence of *t*_σ_ on **r**, for brevity.

#### 2.1.2 The Function Θ_σ_

To make further progress, we define the function
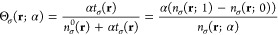
7This function plays a central role in our derivation, therefore we describe it here in detail. Focusing on low values of α (which are particularly relevant for describing the plateau in the KSP potential), and on asymptotically large values of *r* (≡ |**r**|), we realize that for *r* → ∞ the function Θ_σ_(**r**; α) approaches the value of 1, since *t*_σ_(**r**) decays slower than *n*_σ_^0^(**r**).^58^ At somewhat lower values of *r*, however, the term *n*_σ_^0^(**r**) prevails over *αt*_σ_(**r**), as α is small, and then Θ_σ_(**r**; α) drops to 0.

To illustrate the properties of the function Θ_σ_(**r**; α), consider first the exponential asymptotic form for the densities,^59−65^
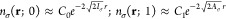
8(a more precise form is discussed below, in eq 11). Substituting these expressions into eq 7, we find that for large values of *r* and for low values of α
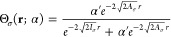
9where . Further algebraic manipulations yield
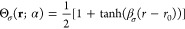
10where  and . A detailed derivation is provided in Appendix A.

**Figure 1:**
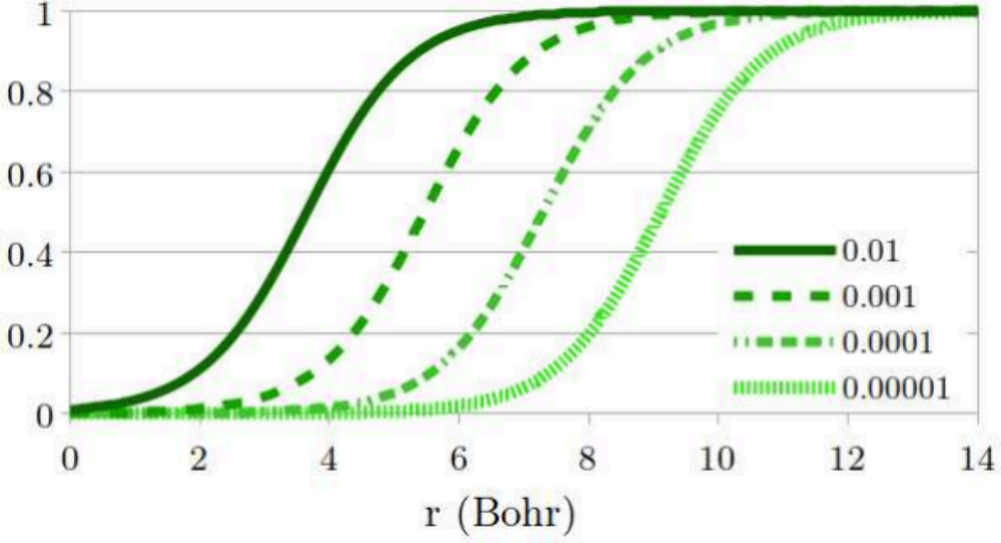
Illustration of the function Θ_σ_(*r*; α), as in eq 10, versus *r*, for β_σ_ = 0.632 a.u., *C*_1_/*C*_0_ = 1 and different values of α (see legend).

**Figure 2:**
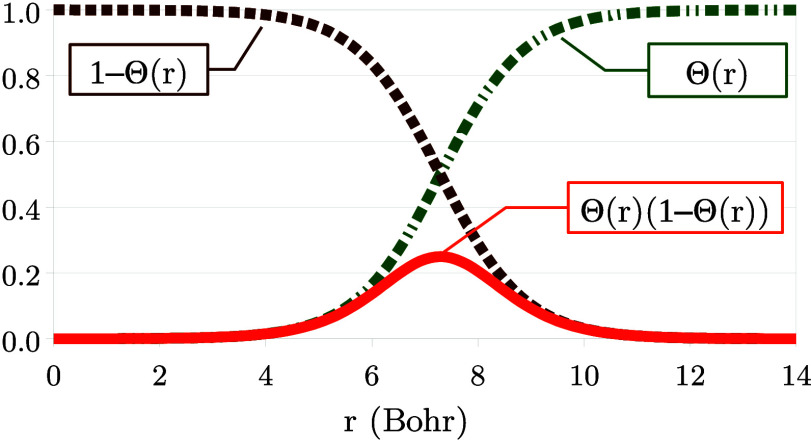
Illustration of the functions Θ_σ_(*r*; α), (1 – Θ_σ_(*r*; α)) and Θ_σ_(*r*; α) (1 – Θ_σ_(*r*; α)) for β_σ_ = 0.632 a.u., *C*_1_/*C*_0_ = 1 and α = 0.0001.

Figure 1 illustrates the dependence of Θ_σ_(**r**; α) on *r* in the asymptotic regime, for various values of α. The horizontal shift in Θ_σ_(**r**), *r*_0_, depends on α logarithmically: the smaller α is, the further the tanh function is shifted away from the origin. Furthermore, in Figure 2 one can see the functions Θ_σ_, (1 – Θ_σ_) and Θ_σ_(1 – Θ_σ_). From Figure 2 we realize that an expression multiplied by Θ_σ_ is relevant in the *far region* (*r* ≫ *r*_0_), an expression multiplied by (1 – Θ_σ_) is relevant in the *near region* (*r* ≪ *r*_0_), and whatever is multiplied by Θ_σ_(1 – Θ_σ_) is relevant in the *intermediate region* (*r* ≈ *r*_0_). These regions depend on the value of α: they drift away from the origin as α → 0^+^. This realization is crucial in the following explanations, as we will express quantities as sums of terms relevant in these three regions.

For future use we bring here also an expression for Θ_σ_(**r**; α), which arises from a more precise assessment of the asymptotic decay of the density. For large *r*, the exact many-electron spin-densities behave as^10,55,63,66^
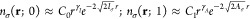
11Here,  and . Then,
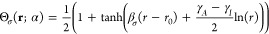
12(see Appendix A for details).

#### 2.1.3 Kohn–Sham–Pauli Plateau in Terms of Θ_σ_

Equipped with the results and intuition for the function Θ_σ_(**r**; α) developed in the previous section, we now apply it, in its exact form, as expressed in eq 7, to the KSP potential of eq 6. For the second and the third terms on the rhs of eq 6, we examine each of the summands that appear in the numerators separately, and obtain
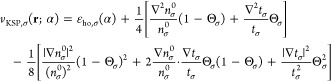
13Using the fact that
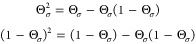
14we rewrite eq 13 as follows:
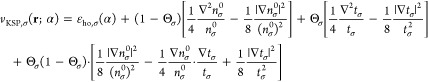
15Interestingly, from this expression we see that the dependence of *v*_KSP,σ_ on α is only via ε_ho,σ_(α) and Θ_σ_(**r**; α).

To make further progress toward the plateau function (eq 2), we introduce a new auxiliary quantity, . Then, we express *t*_σ_, as well as its gradient and Laplacian in terms of τ_σ_ and *n*_σ_^0^. Furthermore, we find that , which allows us to express the gradient and the Laplacian of τ_σ_ in terms of Θ_σ_. Details are provided in Appendix B. The KSP potential can therefore be expressed as
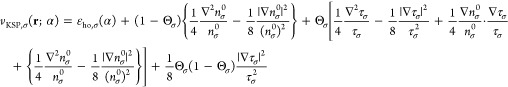
16Focusing on the expression in the curly brackets and comparing it to eq 4, we realize that it is equivalent to *v*_KSP,σ_(**r**; 0^–^) – ε_ho,σ_(0^–^). This allows us to arrive at the plateau function *P*_KSP,σ_(**r**; α) defined in eq 2:
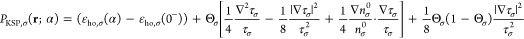
17For the exact case, ε_ho,σ_(α) does not depend on α, as we already mentioned above, and (ε_ho,σ_(α) – ε_ho,σ_(0^–^)) = *I*_σ_ – *A*_σ_ is the σ-fundamental gap of the system. Furthermore, using the relation between τ_σ_ and Θ_σ_, we can express the KSP plateau in terms of *n*_σ_^0^, of Θ_σ_ and of the eigenvalues alone:
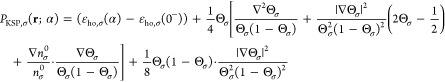
18An equivalent, alternative expression for the plateau is
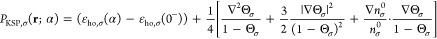
19This concludes our derivation of the exact KSP plateau: we obtained an exact analytical expression for the plateau function *P*_KSP,σ_(**r**; α), which depends on the fundamental gap, the ground-state density *n*_σ_^0^ ≡ *n*_σ_(**r**; 0) and on the function Θ_σ_. Knowledge of the densities *n*_σ_(**r**; 0) and *n*_σ_(**r**; 1) yields the plateau exactly. Properties of the exact plateau *P*_KSP,σ_(**r**; α) in the asymptotic regime are derived below, in Sec. 2.1.4.

#### 2.1.4 Asymptotic Form for the Kohn–Sham–Pauli Plateau

Equations 18 and 19 do not transparently show that the function *P*_KSP,σ_(**r**; α), in the limit α → 0^+^, forms a spatially uniform plateau around the origin and drops to zero far away. To show that this is the case, we start with the approximate form for Θ_σ_, eq 12, and obtain ∇Θ_σ_, |∇Θ_σ_|^2^,  and ∇^2^Θ_σ_. Recalling that  and using eq 14, we arrive at an explicit expression for the plateau *P*_KSP,σ_(**r**; α).
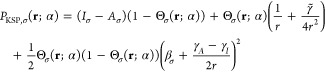
20where . A detailed derivation is provided in Appendix C.

Equation 20 provides us exactly the picture we expected: the plateau in the KSP potential is of the height of the fundamental gap, in agreement with ref (54), and it drops to zero due to the factor (1 – Θ_σ_). The step width, i.e. the typical length scale for the plateau to drop from its maximal height to zero comes directly from the properties of Θ_σ_: it is proportional to 1/β_σ_. In addition, the asymptotic decay of the plateau is 1/*r*, which is the leading term far away (multiplied by Θ_σ_). Finally, in the intermediate region there is a peak, whose height is roughly proportional to β_σ_^2^. It is essential to use the more elaborate asymptotic expressions for the densities, eqs 11 and 12, which include also the factors  and , to obtain the correct ∼1/*r* decay of the plateau function in the far region. A graphical illustration of the KSP plateau and its ingredients is given and discussed in detail in Sec. 4.2.

### 2.2 Pauli Plateau

We now proceed to derive the plateau function for the Pauli potential, *P*_P,σ_(**r**; α) = *v*_P,σ_(**r**; α) – *v*_P,σ_(**r**; 0^–^). The exact expression for *v*_P_(**r**), in terms of the KS orbitals and eigenvalues,^12,54^ plays a crucial role in the derivation. We start with the general form for the Pauli potential for a spin-dependent system with a varying, possibly fractional number of electrons:^54^
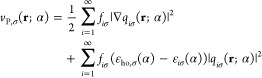
21where *q*_*iσ*_(**r**; α) = φ_*iσ*_(**r**; α)/*n*_σ_^1/2^(**r**; α) is the ratio of the *iσ*^th^ KS orbital and the square root of the σ-density, and ε_*iσ*_(α) and *f*_*iσ*_ are the KS energy levels and occupations, respectively.

For *N*_σ_ = *N*_σ_^0–^, the occupations are
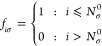
22Using the fact that
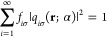
23we express the Pauli potential as
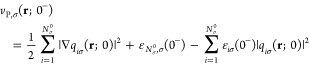
24For *N*_σ_ = *N*_σ_^0^ + α, when the occupations are
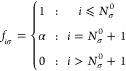
25the Pauli potential can be expressed as
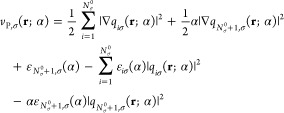
26In the following, we express the Pauli potential for a system with *N*_0_ + α electrons, *v*_P,σ_(**r**; α), in terms of quantities of the *N*_0_-system (i.e., α = 0^–^), which will make it possible to identify *v*_P,σ_(**r**; 0^–^) there, and then obtain the Pauli plateau function.

First, we wish to express the KS eigenvalues as ε_*iσ*_(α) = ε_*iσ*_(0^+^) + *Δε*_*iσ*_(α) = ε_*iσ*_(0^–^) + Δ + *Δε*_*iσ*_(α), where *Δε*_*iσ*_(α) is the change in the *iσ*^th^ KS eigenvalue that occurs as α rises from 0^+^ to a certain positive, finite, value of α, and Δ is the famous derivative discontinuity (eq 1), which is the amount by which all the KS eigenvalues jump as the number of electrons surpasses an integer value (i.e., Δ = ε_*iσ*_(0^+^) – ε_*iσ*_(0^–^)). Incorporating the above expressions into eq 26 and using eq 23 together with eq 25, we find that all the terms multiplied by Δ are eliminated, and the Pauli potential reads:
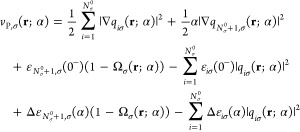
27where
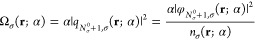
28For the exact KS potential, the ho eigenvalue must not vary at all with the number of electrons: for α ∈ (0, 1], ε_*N*_σ_^0^+1,σ_(α) = −A_σ_ and therefore *Δε*_*N*_σ_^0^+1,σ_(α) = 0. However, for approximate KS potentials, there may be a spurious α-dependence, which is why we include the term with *Δε*_*N*_σ_^0^ + 1, σ_ in the derivation, for now.

Next, we examine in detail the quantity Ω_σ_(**r**; α) of eq 28. Since the density can be expressed as *n*_σ_(**r**; α) = ∑_*i*=1_^*N*_σ_^0^^|φ_*iσ*_(**r**; α)|^2^ + α |φ_*N*_σ_^0^+1,σ_(**r**; α)|^2^, it follows from eq 28 that
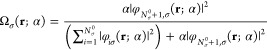
29KS orbitals decay as , and therefore we realize that the quantity Ω_σ_(**r**; α) approaches 1 at infinity and 0 near the origin, similarly to Θ_σ_(**r**; α). In a sense, Ω_σ_(**r**; α) is the KS analog of Θ_σ_(**r**; α): recall that the density *n*_σ_(**r**; α) for a system with a fractional *N* is an ensemble density of a many-electron interacting σ-system, i.e. it equals *n*_σ_(**r**; α) = (1 – α)· *n*_σ_^0^(**r**) + *αn*_σ_^1^(**r**) (cf. eq 5), and it is simultaneously an ensemble density of the corresponding KS system, therefore it can be expressed, without any approximation, as *n*_σ_(**r**; α) = (1 – α)· ρ_σ_^0^(**r**; α) + *αρ*_σ_^1^(**r**; α), where ρ_σ_^*k*^(**r**; α) ≔ ∑_*i*=1_^*N*_σ_^0^+k^|φ_*iσ*_(**r**; α)|^2^.^39,40,54,67^ Then, noticing that ρ_σ_^1^(**r**; α) – ρ_σ_^0^(**r**; α) = |φ_*N*_σ_^0^+1,σ_(**r**; α)|^2^, we can express Ω_σ_ as
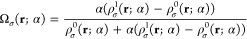
30(cf. eq 7). Furthermore, using the fact that ρ_σ_^0^(**r**; 0) = *n*_σ_^0^(**r**), we express the density *n*_σ_(**r**; α) as (cf. eq 5)
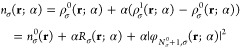
31where  is named the relaxation term, and it measures how much the quantity ρ_σ_^0^ changes with α. We can also define a relaxation term for each KS orbital separately,
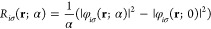
32and then *R*_σ_(**r**;α) = ∑_*i*=1_^*N*_σ_^0^^*R*_*iσ*_(**r**;α). Comparing eqs 31 and 5 we conclude that *t*_σ_(**r**; α) = |φ_*N*_σ_^0^+1,σ_(**r**; α)|^2^ + *R*_σ_(**r**; α) and therefore the quantity Θ_σ_, defined in eq 7, can be expressed as
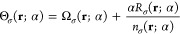
33From the asymptotic behavior of the KS orbitals it follows that for small α the term *αR*_σ_(**r**; α)/*n*_σ_(**r**; α) decays to zero both far from the origin and close to the origin, being relevant (if at all) only in the intermediate region. Therefore, asymptotically Ω_σ_(**r**; α) has just the same behavior as Θ_σ_(**r**; α).

Second, going back to eq 26, we address the quantity |*q*_*iσ*_(**r**; α)|^2^, and express it in terms of the *N*_0_-system.
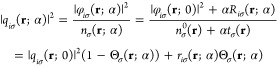
34where *r*_*iσ*_(**r**; α) ≔ *R*_*iσ*_(**r**; α)/*t*_σ_(**r**). In words, as α increases, the quantity |*q*_*iσ*_(**r**; α)|^2^ equals its α = 0-value in the near region, fading out in the far region. In the far region, the relaxation term *r*_*iσ*_ is present.^68^

Furthermore, we note an interesting property for *r*_*iσ*_:
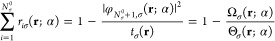
35and therefore at *r* → ∞, ∑_*i*=1_^*N*_σ_^0^^*r*_*iσ*_(**r**; α)→ 0.

Third, still with eq 26 in mind, we proceed to the gradients of *q*_*iσ*_(**r**; α). Using the equality |∇*q*_*iσ*_(**r**; α)|^2^ = |∇(|*q*_*iσ*_(**r**; α)|^2^)|^2^/(4|*q*_*iσ*_(**r**; α)|^2^), which is true as long as the KS orbitals φ_*iσ*_ do not have an **r**-dependent phase (i.e.,∇(arg[φ_*iσ*_(**r**; α)]) = 0), we find that
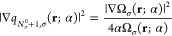
36Furthermore, using eq 34, we find that
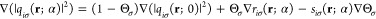
37where *s*_*iσ*_(**r**; α) = |*q*_*iσ*_(**r**; 0)|^2^ – *r*_*iσ*_(**r**; α). For brevity, here and below we drop the argument of Θ_σ_(**r**; α). The quantity *s*_*iσ*_ is close to |*q*_*iσ*_(**r**; 0)|^2^, up to a relaxation term. The square of the modulus of the above gradient equals
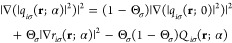
38i.e., in the near region it essentially equals |∇(|*q*_*iσ*_(**r**; 0)|^2^)|^2^, in the far region its behavior is dictated by the relaxation term, and in the intermediate region—by the function
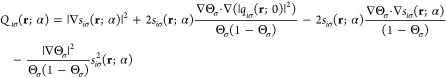
39To obtain the above form for *Q*_*iσ*_ we expressed all the relaxation terms via *s*_*iσ*_(**r**; α).

To express the term |∇*q*_*iσ*_(**r**; α)|^2^, we divide eq 38 by eq 34 and obtain
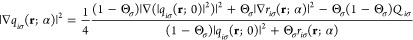
40In eq 40 we see that both the numerator and the denominator are expressed in terms of the near-, far- and intermediate-region expressions. Therefore, in the near region, where Θ_σ_ → 0,  and in the far region, where Θ_σ_ → 1, . The latter may be expressed as  (note that *r*_*iσ*_(**r**; α) is not necessary a positive function). We denote the behavior of |∇*q*_*iσ*_(**r**; α)|^2^ in the intermediate region via the function *W*_*iσ*_(**r**; α), which is determined later on. At this stage, we focus on |∇*q*_*iσ*_(**r**; α)|^2^ and express it as
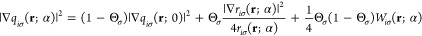
41

Now we are ready to find the expression for the plateau function of the Pauli potential. Inserting eqs 36 and 41 into eq 27 and then subtracting eq 24 from eq 27, according to the definition for the plateau function *P*_P,σ_(**r**; α), and using eq 34, we obtain
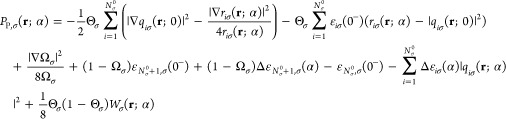
42where *W*_σ_(**r**; α) = ∑_*i*=1_^*N*_σ_^0^^*W*_*iσ*_(**r**; α). Expressing ε_*N*_σ_^0^,σ_(0^–^) as (1 – Θ_σ_)ε_*N*_σ_^0^,σ_(0^–^) + Θ_σ_ε_*N*_σ_^0^,σ_(0^–^) and then focusing on the terms preceded by Θ_σ_, we can recognize there *v*_P,σ_(**r**; 0^–^). Furthermore, presenting the term (1 – Ω_σ_)ε_*N*_σ_^0^+1,σ_(0^–^) as (1 – Θ_σ_)ε_*N*_σ_^0^+1,σ_(0^–^) + (Θ_σ_ – Ω_σ_)ε_*N*_σ_^0^+1,σ_(0^–^), the plateau function obtains the following form:
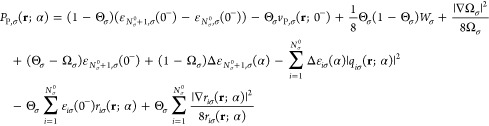
43

Equation 43 is an *exact* expression for the Pauli plateau function. The main features of this plateau are apparent already from eq 43 itself. However, in the following we describe it term-by-term, discuss the significance of each term in the near, far and intermediate regions, and finally produce an approximate, simplified form for the Pauli plateau, in which we choose to focus on the near- and far-region terms, sacrificing exactness in the intermediate region. A graphical illustration of the Pauli plateau, term-by-term, is given in Sec. 4.3 and in more detail in the Supporting Information (SI).

The first term of eq 43 gives the behavior of the plateau in the near region, namely a plateau of the height of the σ-KS gap (*E*_*g*,σ_^KS^ = ε_lu,σ_(0^–^) – ε_ho,σ_(0^–^)), as expected.^54^ The plateau width of the first term, as well as the typical length for it to drop to 0 is dictated by Θ_σ_.

The second term of eq 43 is related to the behavior of the plateau in the far region: there it is dominated by (the negative of) the Pauli potential, – *v*_P,σ_(**r**; 0^–^), which exponentially decays as *r* → ∞(see eq 24). Therefore, also in the far region the contribution of second term is expected to be insignificant.

The ninth term of eq 43, , which is potentially relevant in the far region, also decays exponentially at large *r*. This is because for *i* ⩽ *N*_σ_^0^, as *r* → ∞, the quantity *R*_*iσ*_ decays faster than *t*_σ_ (cf. eqs 32, 5, and 8), therefore *r*_*iσ*_ decays exponentially. Exponential decay is therefore also expected for the derivatives of *r*_*iσ*_. Combined with the fact that the ninth term includes Θ_σ_, the contribution of this term in the far region is not expected to be significant.

Another term which could be potentially relevant in the far region is the eighth term of eq 43, – Θ_σ_ ∑_*i*=1_^*N*_σ_^0^^ ε_*iσ*_(0^–^)*r*_*iσ*_(**r**; α). However, due to the aforementioned exponential decay of *r*_*iσ*_ in combination with the Θ_σ_ function, this term is not expected to contribute in the far region, either. Actually, the eighth term can be roughly approximated as – Θ_σ_ ε̅_σ_ ∑_*i*=1_^*N*_σ_^0^^*r*_*iσ*_(**r**; α) = ε̅_σ_ (Ω_σ_ – Θ_σ_) (see eq 35), where ε̅_σ_ is some effective average energy. From eq 33 and the subsequent explanation it follows that the eighth term of eq 43 is actually an intermediate-region term.

The sixth term of eq 43 is zero for the exact KS potential, because *Δε*_*N*_σ_^0^+1,σ_(α) = 0. For approximate KS potentials it may differ from zero and hence slightly change the height of the plateau in the near region. In any case, in the limit α → 0^+^, this term vanishes, by definition, both for the exact and for approximate KS potentials.

The seventh term of eq 43, ∑_*i*=1_^*N*_σ_^0^^*Δε*_*iσ*_(α)|*q*_*iσ*_(**r**; α)|^2^, vanishes in the limit α → 0^+^, by definition. For finite values of α this term can be bound from above and from below. Since |*q*_*iσ*_(**r**; α)|^2^ ⩾ 0 and ∑_*i*=1_^*N*_σ_^0^^ |*q*_*iσ*_(**r**; α)|^2^ = 1 – Ω_σ_(**r**; α), we can claim that
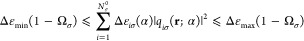
44where *Δε*_min_(α) and *Δε*_max_(α) are respectively the smallest and the largest of all *Δε*_*iσ*_(α). Therefore, the seventh term of eq 43 belongs to the near region and can potentially contribute to the height of the plateau there for finite α. In the limit α → 0^+^ the whole term vanishes, as mentioned above.

The fifth term of eq 43 is an intermediate-region term, because it is preceded by (Θ_σ_ – Ω_σ_)(see eq 33).

The fourth term of eq 43, , is an intermediate-region term. To show this, recall the analogy between Ω_σ_ and Θ_σ_ outlined above (see eq 29 and the explanation there). Thus, Ω_σ_ can be described in a way similar to eq 10 for Θ_σ_, and thus it can be shown that the fourth term of eq 43 decays both in the far region and in the near region, remaining relevant only in the intermediate region.

The last term we discuss is the third term of eq 43, . We aim to show that this is an intermediate-region term, as well. We start with eqs 40 and 41 and find that
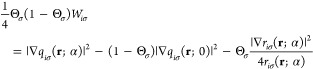
45We note in passing that the last term on the rhs of the above equation exactly cancels out the ninth term of eq 43. For this reason, in the following sections the third and the ninth term will be visualized together.

Focusing on eq 45, after some algebraic manipulations, using again eq 14, we realize that the rhs of eq 45 can be expressed as a fraction where all the terms in the numerator have the prefactor Θ_σ_(1 – Θ_σ_). This allows to express *W*_*iσ*_ as *N*_*W*_/[(1 – Θ_σ_)|*q*_*iσ*_(**r**; 0)|^2^ + Θ_σ_*r*_*iσ*_(**r**; α)], where the numerator *N*_*W*_ reads:
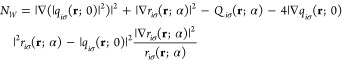
46

Up to this point in the derivation, we presented exact results. However, to make further progress and to arrive at a useful expression for *P*_P,σ_, we leave only the leading terms in the expression for *Q*_*iσ*_ (eq 39) and for *N*_*W*_ (eq 46), taking all the *r*_*iσ*_-dependent terms to zero. Then,
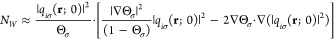
47and
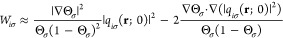
48We recall that ∑_*i*=1_^*N*_σ_^0^^ |*q*_*iσ*_(**r**; 0)|^2^ = 1, and thus ∑_*i*=1_^*N*_σ_^0^^ ∇(|*q*_*iσ*_(**r**; 0)|^2^) = 1, and find that *W*_σ_ = |∇Θ_σ_|^2^/(Θ_σ_(1 – Θ_σ_)^2^). Therefore, the contribution of the third term of eq 43 to the Pauli plateau function is
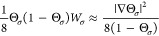
49The derivation for |∇Θ_σ_|^2^ provided in Appendix C helps to see that (49) is indeed an intermediate term.

Finally, relying on the analysis above, we obtain an approximate form for the Pauli plateau function, *P*_P,σ_(**r**; α), in the limit of α → 0^+^. In this limit, we disregard the relaxation terms *r*_*iσ*_(**r**; α) and *Δε*_*iσ*_(α). As a result, the sixth to the ninth terms of eq 43 vanish. Furthermore, from eq 33 it follows that now Ω_σ_ ≈ Θ_σ_, which removes the fifth term of eq 43 and allows to algebraically combine the fourth and the approximate third term (eq 49). This results in the following approximate expression for the Pauli plateau:
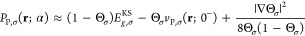
50In the near region we obtain a plateau of the height of the KS gap, whose form is governed by the function Θ_σ_, and in the far region the Pauli plateau contributes a term that decays exponentially. Notably, in the regime when eq 12 is valid, the intermediate region term of the Pauli plateau exactly equals the intermediate term of the KSP plateau (cf. eq 20).

### 2.3 The Spin-Independent Case

Our derivation so far was carried out in the spin-polarized formalism. In this Section, we shortly address the spin-unpolarized case, for completeness.

Usually, spin-unpolarized KS DFT results can be obtained from the spin-polarized case by setting , and consequently *v*_KS,↑_(**r**) = *v*_KS,↓_(**r**), ε_*i*,↑_ = ε_*i*,↓_ and φ_*i*,↑_(**r**) = φ_*i*,↓_(**r**), as expected. We cannot directly do so in this derivation, because we considered so far the common case when the occupation numbers change only in one of the spin channels. Hence, we cannot require that *f*_*i*,↑_ = *f*_*i*,↓_. In this Section, we therefore explicitly analyze the spin-independent case, focusing on the scenario when the *N*_0_-system is a closed-shell system, and the (N_0_ + 1)^th^ electron is added to a previously vacant state, in correlation with the spin-unpolarized numerical results we present below.

For the KSP potential, in the spin-unpolarized case we write the fundamental eq 3 in the same manner, just without the index σ. Consequently, all the results for the KSP potential and plateau are completely analogous to those presented in Sec. 2.1.

As to the Pauli potential, in the aforementioned scenario of *N*_0_ being a closed-shell system, the occupation numbers are
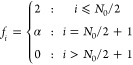
51where all the fully occupied levels hold two electron each, and the (N_0_/2 + 1)^th^ level is fractionally occupied with α electrons (cf. eq 25). Furthermore, note that the quantity *q*_*i*_(**r**; α) = φ_*i*_(**r**; α)/(*n*^1/2^(**r**; α)) equals its spin-polarized analogue when  is substituted, up to a factor of √2. Yet, eqs 23 and 28 have direct analogues in our spin-unpolarized scenario and are obtained by dropping everywhere the index σ and substituting *N*_σ_^0^ by *N*_0_/2. Other equations, particularly eq 43, are translated to our case by the following three actions: dropping everywhere the index σ, substituting
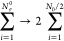
52and substituting *N*_σ_^0^ elsewhere by *N*_0_/2. All the conclusions about the various terms of eq 43, their regions of relevance and approximation, are relevant in the spin-polarized case, as well.

## 3 Numerical Details

For results presented in Secs. 4.1–4.4, the exact ground-state densities were obtained with the FCI method, as in refs (50) and (54): For integer *N*, the many-electron problem was solved using MOLPRO^69^ in the Universal Gaussian Basis Set^70^ and the density was obtained from the FCI wave function using ORBKIT^71^ – a python package for postprocessing wave function data. All FCI results are spin-unpolarized.

The resulting exact densities were then treated in two alternative manners, with the aim of checking numerical accuracy and stability. (A) The densities were extrapolated to correct the asymptotic inaccuracy in the low density region of the FCI solution.^72^ This was done by detecting the point where the density decays exponentially as in eq 8, i.e., the logarithm of the density decays linearly, and continuing this decay for all further points. For Li, this point is at 10 Bohr, and for Li^+^ at 2.6 Bohr (see the Supporting Information of ref (50) for more details). This extrapolation resulted in an exponential behavior of the densities far from the system center, but introduced some shortcomings. First, there is a small inconsistency between the IP of the system as obtained from total-energy differences and the IP that can be determined from the asymptotic decay rate. Second, the second derivative of the densities is discontinuous at the stitching points. Third, the power-law dependence in the density decay (eq 11) is not recovered. Therefore, we could not evaluate the accuracy of the approximation for Θ_σ_ presented in eq 12 vs eq 10, because the former is based on eq 11.

An alternative route was proceeded, as well. (B) The Li^+^ density was fit to the function *n*_Li^+^_(*r*) = *A*[*e*^–α_1_*r*^ + *b*_1_*e*^–α_2_*r*^], with α_1_ = 4.8135, α_2_ = 7.16 and *b*_1_ = 1.02. The coefficient *A* = 6.7753 was obtained by normalizing the density function to 2 electrons. The Li density was fit to *n*_Li_(*r*) = *C* [(1 + *c*_1_*r*^2^) *e*^–γ_1_*r*^ + *d*_1_*r*^2^*e*^–γ_2_r^ ], with γ_1_ = 5.936, γ_2_ = 1.3202, *c*_1_ = 0.575, and *d*_1_ = 0.000976. The coefficient *C* = 13.81944 was obtained from normalization to 3 electrons. The exponential decay of the fit functions removes the deviation of the FCI from the exponential decay regime at low densities, while keeping the resultant density smooth to all orders. The optimization of the parameters was carried out such that the resultant Θ-function always remains positive. Furthermore, the fit detailed above made it simpler to present densities on a real-space grid. Results from both approaches, (A) and (B), yielded densities, Θ- and Ω-functions in close correspondence to each other, except when analyzing the approximate asymptotic forms for the Θ-functions, as in eq 12 vs eq 10, where Approach (B) was advantageous. Hence, it was used also for the following numerical steps.

Densities for systems with fractional *N* were calculated as a linear combination of the pure-state densities discussed above, as in eq 5. Then, to obtain the corresponding KS potential, orbitals and eigenvalues, we performed a numerical inversion using the ORCHID all-electron real-space spherical atomic code.^73,74^ The calculations were performed on a natural logarithmic radial grid of 16,000 points, for *r* ∈ [*e*^*c*^/*Z*, *L*], with *c* = −13, *L* = 35 Bohr and *Z* being the atomic number. The inversion method is that of ref (75) (see details also in ref (31), Sec. 3). Within this method, the inverted KS potential is searched iteratively, following the formula *v*_KS_^(*k*+1)^(**r**) = *v*_KS_^(*k*)^(**r**) + μ{[*n*^(*k*)^(**r**)]^*p*^ – *n*_target_(**r**)]^*p*^}, where the superscripts denote the iteration number, i.e., *n*^(*k*)^(**r**) is the density obtained at the *k*th iteration, and *n*_target_(**r**) is a preset target density, which the inverted potential has to reproduce. We used the parameter *p* = 0.05 to account for changes in the potential at far distances, and μ varied to ensure rapid convergence. The convergence criterion for the inversion procedure is ln(*n*(*r*)/*n*_target_(*r*)) < 3 · 10^–4^, enforced for *r* ∈ [0.005, 29]Bohr. The inverted potentials were found to decay as ∼ *a*/*r* + *b*, and were aligned at infinity by a linear fit of the potential vs 1/*r* at 18.01 and 28.00 Bohr.

For results of Sec. 4.5, the LSDA calculations were performed with the ORCHID atomic code, on a natural logarithmic radial grid of 13.000 points, for *r* ∈ [*e*^*c*^/*Z*, *L*], with *c* = −13, *L* = 25 Bohr. All these calculations were spin-polarized, namely the added electron was introduced to a specific spin channel, as mentioned in Sec. 2.1.

Finally, note that in Figures 5 and 7 presented below in Sec. 4 we demonstrate full overlap between certain quantities, by plotting one of them with a thick colored line and the other one with a thin *white* line, above the colored one. Perfect overlap thus creates a hollow, spaghetti-like line. This graphical approach served us well in the past,^54,67^ and allowed to concisely present several quantities, some of which are overlapping. In addition, the fact of a full overlap is mentioned in text and in the captions of these figures.

## 4 Computational Results

### 4.1 The Θ Function and Its Approximations

To illustrate the analytical results derived in Sec. 2, we numerically examine the Li atom with 2 + α electrons, namely the transition from Li^+^ to Li. We rely on exact FCI densities, obtained in a spin unpolarized manner, as detailed in Sec. 3.

**Figure 3:**
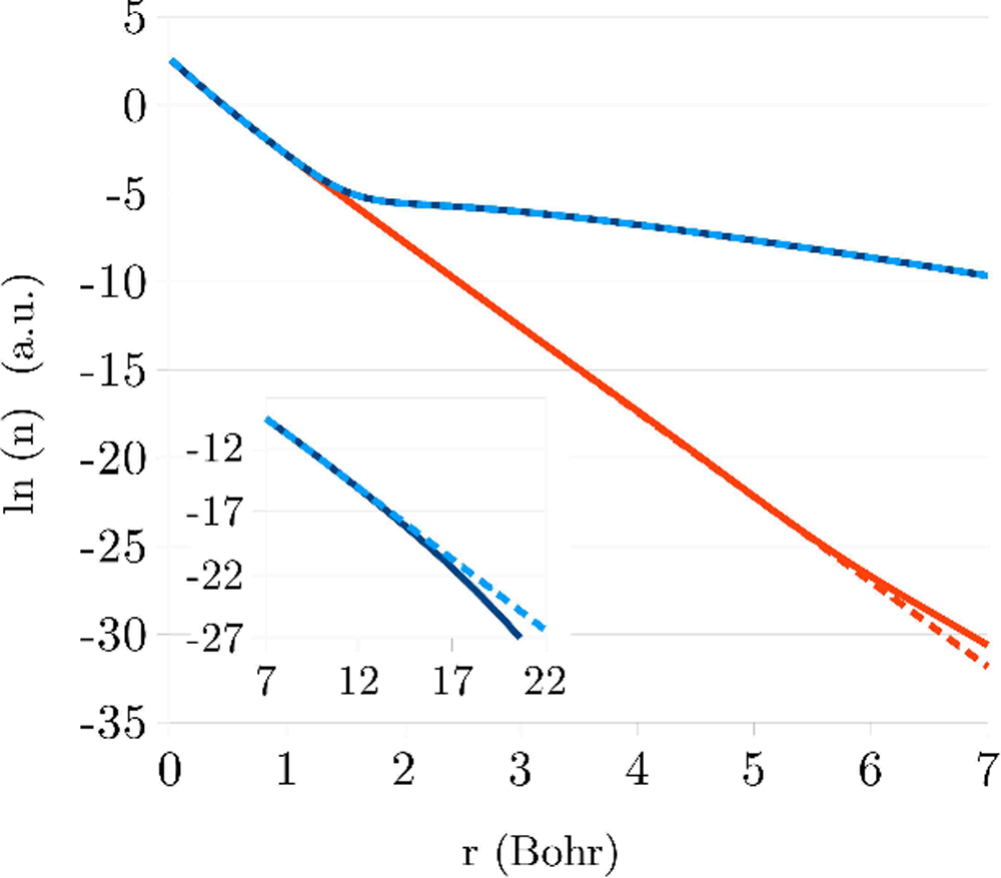
Logarithm of the densities of neutral (blue) and cationic (orange) Li, obtained from FCI calculations. Solid lines: raw FCI data, dashed lines: fitted functions, as described in Sec. 3. Discrepancy for the cation is visible beyond 5 Bohr, and for the neutral—beyond 15 Bohr (see inset).

The FCI densities for Li^+^ and Li are depicted in Figure 3 on a logarithmic scale, along with their analytic fits (see Sec. 3). The different asymptotic behavior of the cationic and the neutral densities can be observed. Furthermore, the close correspondence of the fits to the original data, as well as the point of their bifurcation, are clearly seen.

**Figure 4:**
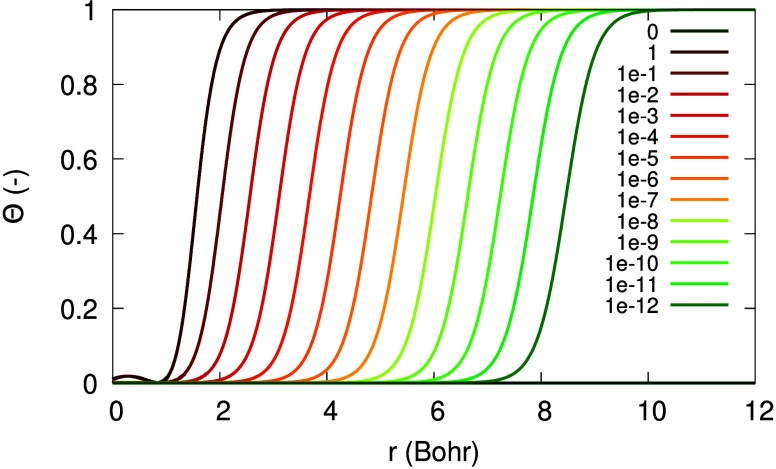
Function Θ(*r*; α), obtained from FCI calculations for the Li atom with 2 + α electrons (see legend).

The function Θ(*r*; α) obtained from the fitted FCI densities is shown in Figure 4, for different values of α. As expected, Θ(*r*; α) approaches 1 at large values of *r* and 0 near the origin. Furthermore, as α → 0^+^, the function Θ(*r*; α) travels away from the origin at a logarithmic pace: the point *r*_0_ (for which ) depends on α logarithmically.

**Figure 5:**
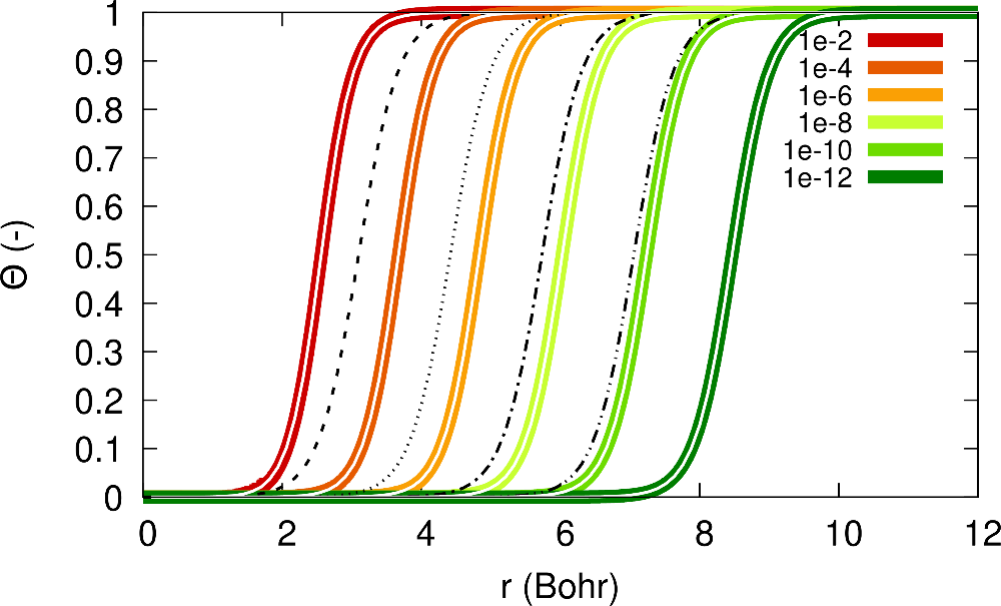
The exact function Θ(*r*; α), obtained from FCI calculations for the Li atom with 2 + α electrons (see legend). Black: the approximation Θ^(10)^(*r*; α) (as in eq 10), for α = 10^–2^ (dashed), α = 10^–4^ (dotted), α = 10^–6^ (dash-dot), α = 10^–8^ (dash-dot-dot). White: the approximation Θ^(12)^(*r*; α) (as in eq 12, full overlap with colored graphs is observed).

Next, in Figure 5 we examine two approximations for the function Θ(*r*; α): the first is the one given in eq 10 and is denoted here Θ^(10)^(*r*; α) and the second is the one from eq 12, which is denoted Θ^(12)^(*r*; α). The parameters β, *r*_0_, γ_*I*_ and γ_*A*_ were deduced directly from the fit parameters for the density functions. We find that while Θ^(10)^(*r*; α) correctly reproduces the main features of the exact Θ, it travels away too fast, while Θ^(12)^(*r*; α) reproduces the exact Θ very accurately: it completely overlaps with the exact result.

**Figure 6:**
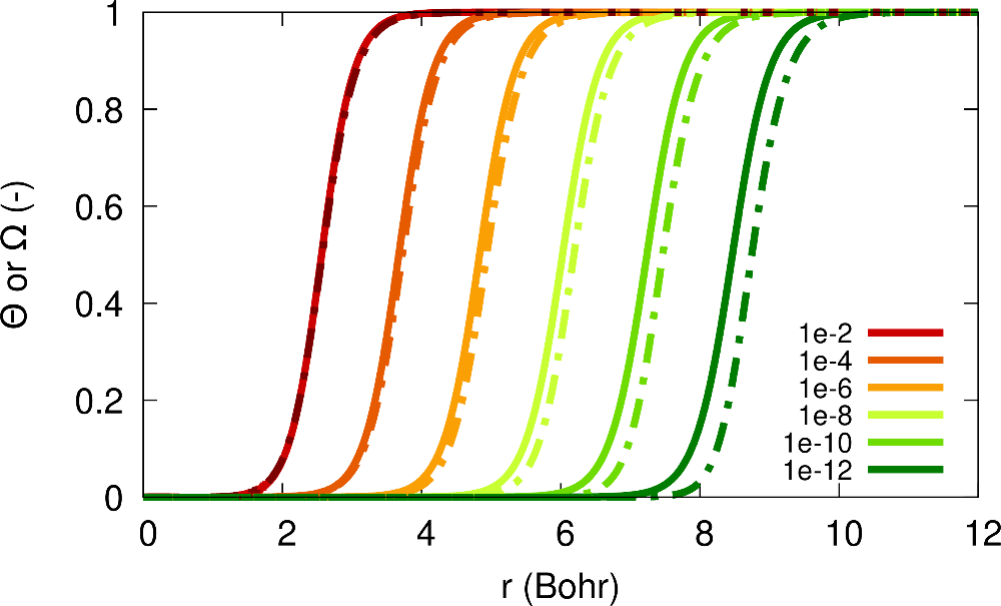
Solid lines: The exact function Θ(*r*; α), obtained from FCI calculations for the Li atom with 2 + α electrons (see legend). Dashed lines: same, for Ω(*r*; α)

Comparison of the exact Θ(*r*; α) and Ω(*r*; α) is shown in Figure 6. As expected from analytical considerations, these functions are rather similar in nature, both approach 1 at infinity and 0 near the origin, and differ only in the intermediate region. This difference becomes more significant, though, as α decreases. Figure S1 in the SI depicts also the difference Θ(*r*; α) – Ω(*r*; α) and shows that this difference can be approximated by *K*(α) Θ(*r*; α)(1 – Θ(*r*; α)), thus illustrating that Θ – Ω is an intermediate-region term. Finally, Figure S2 in the SI numerically demonstrates eq 33, relating the difference Θ – Ω to the relaxation term *R*(*r*; α).

### 4.2 The KSP Plateau Function

**Figure 7:**
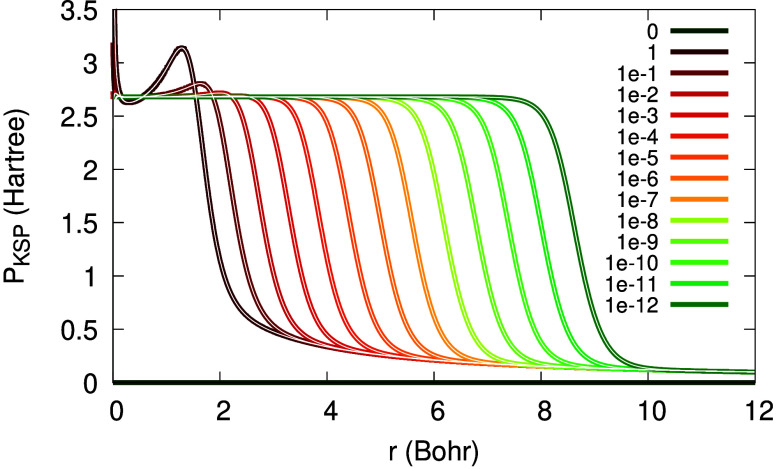
Colored: the KSP plateau *P*_KSP_(*r*; α) from eq 19, for the Li atom with 2 + α electrons, for various values of α (see legend). White: the exact KSP plateau obtained from KSP potential differences (full overlap with the colored graphs).

After obtaining Θ(*r*; α), we are ready to calculate the KSP plateau relying on the analytical results of Sec. 2. First, in Figure 7 we examine the KSP plateaus obtained from eq 19 versus the exact results, namely KSP plateaus directly obtained from potential differences, *v*_KSP_(*r*; α) – *v*_KSP_(*r*; 0^–^). This serves as a sanity check for our analytic derivation, because no assumptions whatsoever were made to obtain eq 19. Indeed, the results coincide to a high degree.

The graphs in Figure 7 present the features expected of a plateau function: They start at a certain height and are almost flat in the near region, especially for α < 10^–2^ (for higher α-values, we observe a typical maximum (see, e.g.^31,48,76^ and references therein), just before the decrease in the plateau height). At a certain distance from the origin, dictated by the value of α, a rapid drop in the plateau height occurs, and finally a decay toward 0 is observed for all α values.

Notably, to get the KSP plateau of eq 19, no information regarding the KS eigenvalues is required, despite the fact that such eigenvalues explicitly appear in eq 19. The way around is plotting the expression in the second line of eq 19, which depends only on Θ and *n*^0^. This expression decays to the same value, for all values of α, which is the fundamental gap, *E*_*g*_. To accurately find *E*_*g*_, one can, for example, plot the aforementioned expression versus 1/*r* and find that there exists a region of linear dependence on 1/*r* (i.e., *P*_KSP_ decays as *r*^–1^). A linear fitting of this region yields the value of 2.689 hartree for the plateau height, which is in close correspondence to ε_ho_(α) – ε_ho_(0^–^) (see Figures S3 and S4 in the SI).

**Figure 8:**
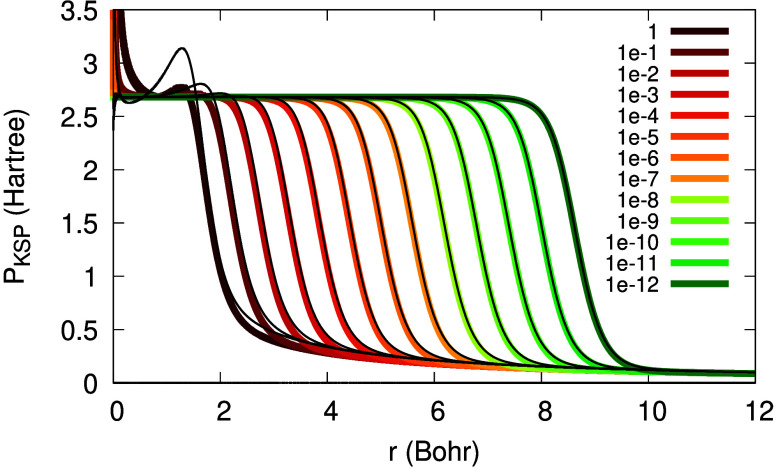
The KSP plateau from eq 20, for the Li atom with 2 + α electrons, for various values of α (see legend). Black: KSP potential differences.

Next, we examine the KSP plateau obtained from the approximate expression in eq 20. Figure 8 presents the KSP plateau versus potential differences. The discrepancies are minor, but visible: they are more significant for high values of α and almost disappear as α →0^+^. This is consistent with the assumption used to derive eq 20: it is valid only asymptotically.

**Figure 9:**
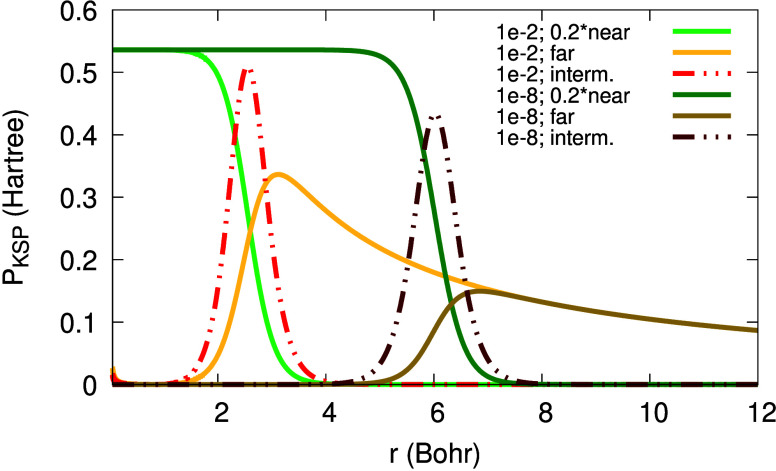
Three terms of the KSP plateau of eq 20 for the Li atom with 2 + α electrons: near-region (solid line; scaled by a factor of 0.2); far-region (dash-dot); intermediate region (dash-dot-dot). All three terms are presented for two values of α: 10^–2^ (bright colors, concentrated around 3 Bohr) and 10^–8^ (dark colors, concentrated around 6 Bohr).

Looking deeper into the approximate KSP plateau of eq 20, we present in Figure 9 its three terms: the near-, the far- and the intermediate-region terms (note that in Figure 9 the near-region term is multiplied by 0.2, for clarity of presentation). We learn from this figure that the near-region term is the dominant one for the plateau construction in Li. The magnitude of the intermediate and far-region terms decreases as α → 0^+^, and furthermore, the far-region term shifts to the right more than the other terms. These two observations may explain why the characteristic maximum in the KSP plateau disappears at low α’s.

### 4.3 The Pauli Plateau Function

**Figure 10:**
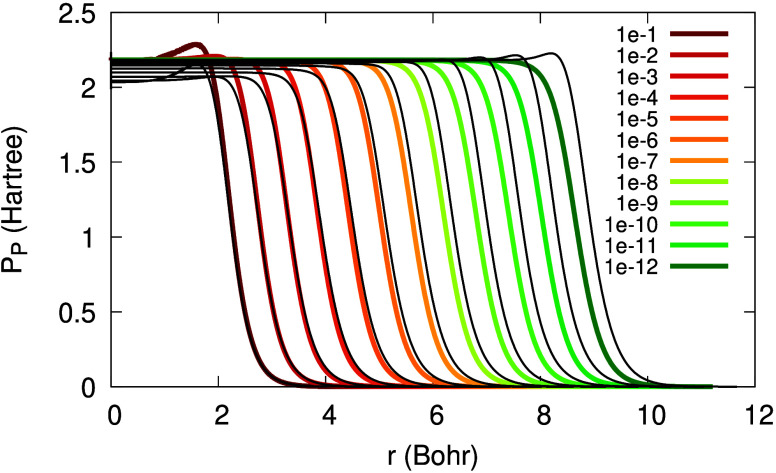
Pauli plateau function of eq 50 (colored) versus the exact Pauli plateau obtained from potential differences (solid black), for the Li atom with 2 + α electrons, for various values of α (see legend).

**Figure 11:**
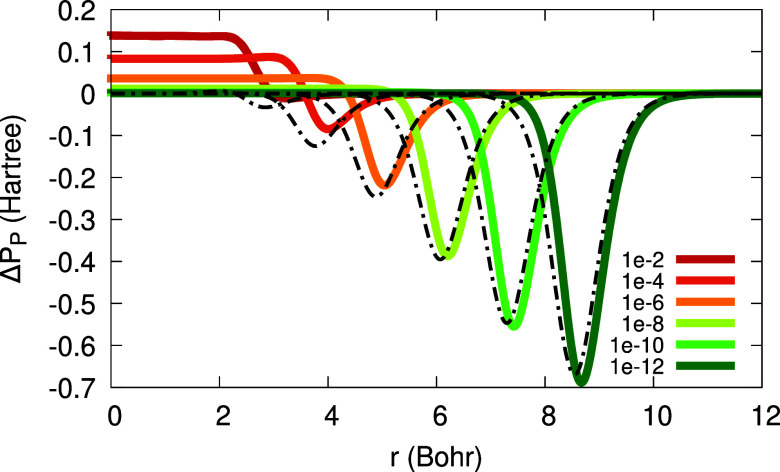
The difference between the Pauli plateau of eq 50 and the exact result from Pauli potential differences, for the Li atom with 2 + α electrons, for various values of α (see legend). Black, dash-dot: the function 2.5 (Ω(*r*; α) – Θ(*r*; α)), for the same α-values, for comparison.

We now examine the approximate Pauli potential plateau of eq 50. Figure 10 presents the Pauli plateau versus the corresponding potential differences. Furthermore, Figure 11 displays the difference between the two (colored graphs). Figures S5 – S14 in the SI show all the nine terms of the exact Pauli plateau (eq 43) and confirm the theoretical statements of Sec. 2.2 as to their properties.

Despite the significant approximations made in the derivation of eq 50 out of eq 43, we find that the description of the Pauli plateau in Li is satisfactory. The height of the plateau at α → 0^+^, the plateau decay and its overall form are accurately reproduced. However, two features distinguish the plateau graphs of eq 50 from the exact ones: (i) In the exact case, the plateau height gradually approaches its asymptotic value as α → 0^+^, whereas in the approximate form the plateau height is constant. This can be directly attributed to the fact that we neglected the seventh term in eq 43. Yet, this discrepancy disappears in the limit α → 0^+^. (ii) As α → 0^+^, plateaus travel away from the center of the system. However, the approximate Pauli plateau “lags behind” the exact one. This deficiency is related to the approximations we made to intermediate-region terms in the derivation of eq 50. Figure 11 shows that at low α values the difference in the Pauli plateaus is entirely and intermediate-term feature: it is proportional to the difference Θ(*r*;α) – Ω(*r*; α) (see Figure S1 in the SI).

### 4.4 The Kohn–Sham Plateau Function

**Figure 12:**
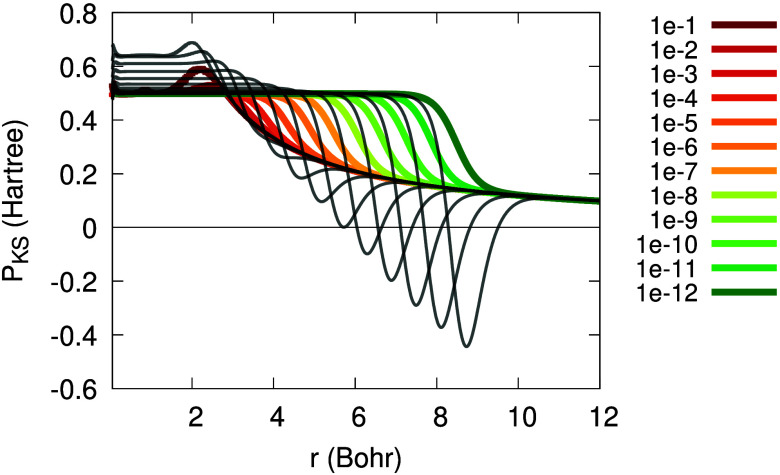
KS plateau functions obtained as in eq 53 (colored) versus the exact KS plateaus obtained from potential differences, for the Li atom with 2 + α electrons, for various values of α (see legend).

**Figure 13:**
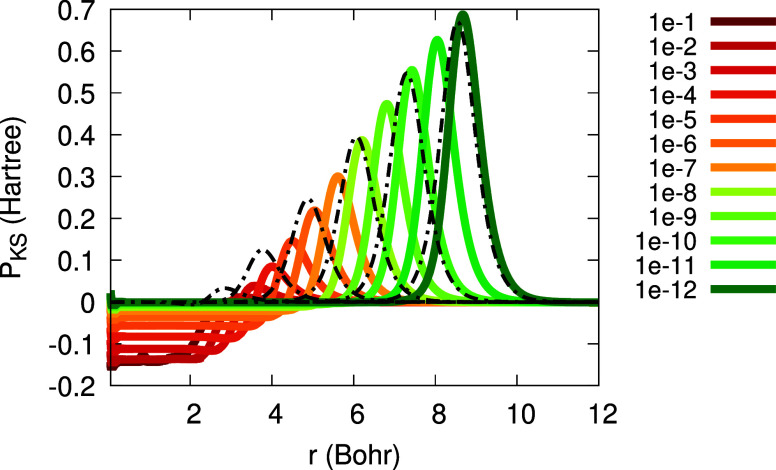
The difference between the KS plateau of eq 53 and the exact KS plateau from potential differences, for the Li atom with 2 + α electrons, for various values of α (see legend). Black, dash-dot: the function −2.5(Ω(*r*; α) – Θ(*r*; α)), for α = 10^–2^, 10^–4^, 10^–6^, 10^–8^, 10^–10^ and 10^–12^ (left-to-right), for comparison.

The plateau function of the KS potential, *P*_KS,σ_(**r**; α) = *v*_KS,σ_(**r**; α) – *v*_KS,σ_(**r**; 0^–^), can be obtained by subtraction of the Pauli plateau from the KSP plateau function,
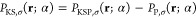
53Figure 12 presents the KS plateau obtained as the difference of the plateaus from eq 19 and eq 50, versus the exact KS plateaus obtained from differences of KS potentials, which emerged from numerical inversion. The discrepancies discussed in the context of the Pauli plateaus are even more apparent here: our approximation completely misses the characteristic dip in the KS plateau that appears in the intermediate region and does not reconstruct the gradual changes in the plateau height. Still, the basic features of the KS plateau in the near and the far regions are captured: a correct plateau height in the α → 0^+^ limit, which equals Δ = *E*_*g*_ – *E*_*g*_^KS^, a logarithmic propagation further from the center of the system and a correct decay at *r* → ∞. Furthermore, as follows from Figure 13, the missing features in the KS plateaus are approximately proportional to Θ(*r*; α) – Ω(*r*; α), which in turn is proportional to Θ(*r*; α)·(1 – Θ(*r*; α)). This information could be important in future attempts to refine the approximation of the KS and Pauli plateaus.

### 4.5 Plateaus with the Local Spin Density Approximation

So far we presented results that were based on exact densities. In this section, we show plateaus that emerge when using approximate densities, which were obtained with the simplest xc approximation, the LSDA, in its standard implementation. To this end, calculations for Li with 2 + α electrons were performed, in a spin-polarized manner, where a fraction of α electrons has been added to a specific (up) spin channel.

The Θ_σ_-function and the Ω_σ_-function obtained with LSDA are qualitatively similar to the exact ones (presented in Sec. 4.1). Figures for these quantities are given in the SI (Figures S15–S17).

**Figure 14:**
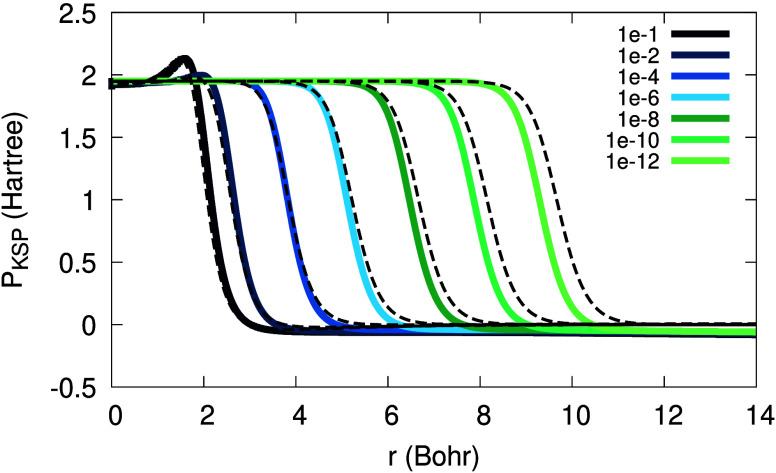
KSP plateau *P*_KSP,↑_(*r*; α) from eq 19, for the Li atom with 2 + α electrons, calculated with LSDA, for various values of α (colored) versus the KSP plateau obtained from potential differences (dashed black).

Figure 14 shows the KSP plateau for Li with LSDA that is calculated using eq 19, versus KSP potential differences. The general features of the plateau function are described in a satisfactory manner. Two shortcomings have to be mentioned, though: (i) The height of the plateau obtained from eq 19 does not equal the true fundamental gap of the system, namely the one obtained from LSDA total energy differences, but it equals the KS gap, due to the inherent lack of a derivative discontinuity in LSDA. (ii) The decay rate of the plateau at far distances is exponential, and is not proportional to *r*^–1^, due to the incorrect decay of the density.

**Figure 15:**
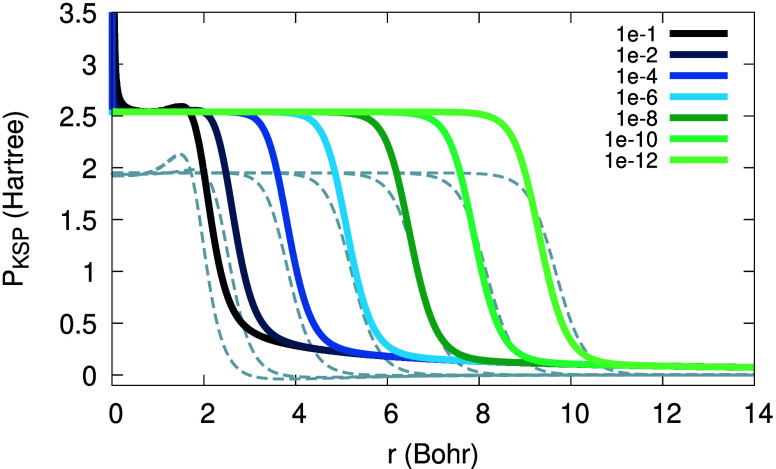
KSP plateau *P*_KSP,↑_(*r*; α) from eq 20, for the Li atom with 2 + α electrons, calculated with LSDA, for various values of α (colored) versus KSP plateaus obtained from potential differences (dashed gray).

In contrast, the plateau that is obtained from eq 20, and is presented in Figure 15, shows a different height and decay rate of the plateau (compare colored and gray curves). The plateau decays as 1/*r*, at least in the interval 13–25 Bohr, and its height equals, by construction, the LSDA fundamental gap, obtained from total energy differences. Therefore, although eq 20 is only asymptotically correct, here it actually helps to construct a more meaningful plateau, even from LSDA results; knowledge of total-energy differences is needed, though.

**Figure 16:**
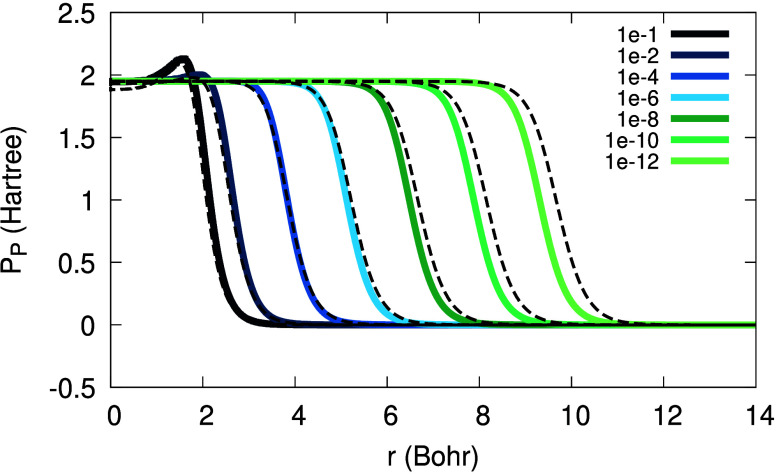
Pauli plateau *P*_P,↑_(*r*; α) of eq 50 for the Li atom with 2 + α electrons, calculated with LSDA, for various values of α (colored) versus the same plateau obtained from potential differences (dashed black).

The Pauli plateaus obtained from eq 50 and from potential differences are presented in Figure 16. The height of the plateau equals the KS gap of the system, as required. As in the exact case, also here we see that our approximation of the Pauli plateau is fair for Li with LSDA, except some expected discrepancies in the intermediate region.

**Figure 17:**
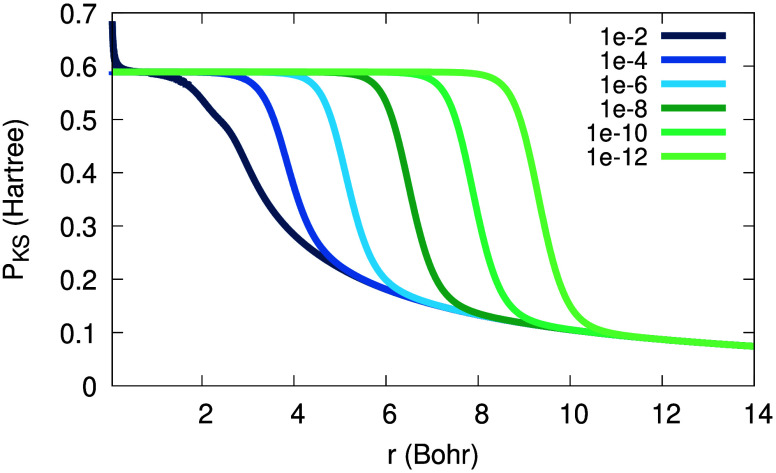
KS plateau function, *P*_KS,↑_(*r*; α) obtained as a difference of eqs 20 and 50, for the Li atom with 2 + α electrons, calculated with LSDA, for various values of α (see legend).

Finally, we wish to obtain a KS plateau, from previously presented results, by subtraction. Trying to do so subtracting the data in Figures 14 and 16 does not yield a meaningful result: the plateau height in these two figures is the same, up to numerical accuracy. However, subtraction of the data in Figures 15 and 16 yields Figure 17. We clearly see the expected characteristics of the KS plateau function: a constant height, logarithmic drift from the system center, 1/*r* asymptotic decay. However, a maximum followed by a dip in the intermediate regions are missing, which should also be expected.

## 5 Summary

In this work, we derived an analytical expression for the plateau that emerges in the Kohn–Sham potential, when varying the number of electrons in the system and surpassing an integer. In our derivation, we formally used the framework of orbital-free DFT, and analyzed the Kohn–Sham–Pauli (KSP) and the Pauli potentials.

In our derivation, we introduced the quantity Θ_σ_(**r**; α), defined in eq 7, which depends on the electron densities with integer number of electrons, *N*_0_ and *N*_0_ + 1, that comprise the ensemble density for intermediate fractional electron number. Using Θ_σ_(**r**; α), an exact expression for the KSP plateau was analytically derived, directly from the central equation of OF-DFT, using the piecewise-linearity property of the ensemble density. Furthermore, both the function Θ_σ_ and the KSP plateau were approximately described in the asymptotic regime. This exposed the behavior of the plateau in the near, far and intermediate regions, and clearly showed which physical quantities govern its height, width and decay rate. Next, the Pauli plateau function has been derived, relying on an exact expression for the Pauli potential in terms of Kohn–Sham quantities (eq 21). First, an exact expression for the Pauli plateau was obtained (eq 43). Each of its components was analyzed, in terms of its contribution to the near, far and intermediate regions. Finally, the exact expression for the Pauli plateau was simplified (eq 50), by neglecting intermediate-region contributions and fixing the height of the plateau to be the KS gap. Finally, the plateau in the KS potential was directly obtained as the difference between the KSP and Pauli plateau functions.

Our derivations were put to test against numerical calculations of plateaus in the Li atom, relying on exact electron densities, obtained from FCI calculations. The resulting plateaus are in close agreement with the exact ones; discrepancies were explained, in view of the assumptions we made in the derivation. Furthermore, it was shown that even when using approximate densities that emerge from LSDA calculations, plateaus in the KSP, Pauli and KS potentials can be obtained.

The present contribution focused on the Li atom, for illustration and benchmarking purposes. Whether the terms neglected and approximated in our treatment are significant in other atoms and molecules and different chemical scenarios, remains a subject of future work.

Incorporation of plateaus in Kohn–Sham DFT calculations, in an exact or approximate manner, e.g., as an *ad hoc* addition to an existing approximate KS potential, is expected to significantly improve the prediction of fundamental gaps, at least for finite systems, and potentially produce better KS energy levels to obey exact properties, such as the IP theorem. Furthermore, a similar analytical approach can be applied to other scenarios where plateaus occur, namely in dissociation and charge transfer. There, it is expected to eliminate the spurious fractional dissociation problem, at any atomic separation, and account for the discrete nature of an electron. In all these scenarios, our approach allows introducing the desired sharp features in the KS potential, at modest computational cost. The latter consideration is particularly important when considering large molecules and when addressing systems that evolve in time, e.g. within real-time time-dependent density functional theory.

## Appendices

### Appendix A Derivation of eqs 10 and 12

We start the derivation from eq 7 of the main text. Using the asymptotic form of the densities (eq 8) and substituting it into eq 7 we get

A1

Multiplying both the numerator and the denominator by  we obtain

A2

where  and . Next, we introduce *r*_0_ such that *e*^–2β_σ_*r*_0_^ = α″. This allows us to simplify the expression for Θ_σ_ to be

A3

To further simplify the expression, we recall the following identities for hyperbolic functions:

A4

Using both, we arrive at the following expression:

A5

Taking the limit of α → 0^+^, we arrive at eq 10 of the main text.

The derivation of eq 12 is obtained in a similar fashion to the above, using the more precise expressions for the densities, given in eq 11. First, we insert the asymptotic expressions of the densities into eq 7:

A6

Next, we multiply both the numerator and the denominator by , and introduce β_σ_ and *r*_0_:

A7

Using the same hyperbolic identities, we obtain eq 12 of the main text. Notably, no assumptions have been made regarding γ_*A*_ and γ_*I*_: their relations to *Z*, *N*_0_, *I*_σ_ and/or *A*_σ_ were not used here.

### Appendix B Detailed Derivation of the KSP Potential

We elaborate here on the derivation of the KSP potential, focusing particularly on the quantity τ_σ_(**r**) and its use in the derivation of the KSP plateau function.

By definition in the main text, . Therefore, dropping in what follows the **r**-dependence for brevity, we can write *t*_σ_ = *n*_σ_^0^τ_σ_, and therefore

B1

B2

B3

and

B4

As a result,

B5

Furthermore, from eq B1 we realize that

B6

and therefore

B7

Next, from eq 7 of the main text we find that in terms of τ_σ_, the function Θ_σ_ can be expressed as

B8

It therefore follows that  and that . Applying the ∇ operator, we find that

B9

B10

B11

B12

and

B13

The relations detailed above are instrumental in the derivation of eqs 16, 17, and 18 of the main text.

### Appendix C Detailed Derivation of the KSP Potential in the Asymptotic Limit

A detailed derivation of eq 20 of the main text is provided below. We start with the asymptotic expression for Θ_σ_ (eq 12 of the main text): , where , , γ = γ_*A*_ – γ_*I*_,  and .

Since Θ_σ_ depends only on the absolute value of **r**, we find that

C1

C2

and

C3

From the expression for the density  we find that

C4

and therefore

C5

Collecting these results together, we get

C6

Next, we notice that tanh(*y*) = 2 Θ_σ_ – 1 and that 4Θ_σ_(1 – Θ_σ_) = (1 + tanh(*y*)) (1–tanh(*y*)) = 1/cosh^2^(*y*). We use these expressions to rewrite eq C6 as follows:

C7

For brevity, let us denote the left-hand side (lhs) of eq C7 by *w*_σ_(**r**; α). Then, eq 19 of the main text reads *P*_KSP,σ_(**r**; α) = (ε_ho,σ_(α) – ε_ho,σ_(0^–^)) + *w*_σ_(**r**; α). We now rearrange terms on the right-hand side (rhs) of eq C7, by powers of Θ_σ_, and find that

C8

We now recall that Θ_σ_^2^ = Θ_σ_ – Θ_σ_(1 – Θ_σ_) to obtain

C9

Next, we rearrange the terms that appear in the square brackets, by powers of 1/*r*. Remarkably, we find that

C10

C11

and

C12

We can therefore express *w*_σ_(**r**; α) as

C13

For the exact xc functional, (ε_ho,σ_(α) – ε_ho,σ_(0^–^)) = (*I*_σ_ – *A*_σ_). This allows to combine the first term from the expression for *w*_σ_(**r**; α) with the first term of *P*_KSP,σ_(**r**; α) in eq 19 (main text), to produce a term that is preceded by the function (1 – Θ_σ_). This concludes our derivation and yields eq 20 of the main text.

Interestingly, as one checks which term of eq 19 (main text) contributes to the term (*I*_σ_ – *A*_σ_)(1 – Θ_σ_), one finds that *all* terms contribute to this expression. Disregarding any of these terms would yield an incorrect result for the plateau function in the near region.

Furthermore, note that using the more elaborate asymptotic expressions for the densities, eqs 11 and 12 (main text), which include also the factors  and , is essential to obtain the correct ∼1/*r* decay of the plateau function in the far region. Otherwise, e.g. when using the regular exponential decay for the densities (eqs 8 and 10), one obtains a plateau that decays as ∼ β_σ_/*r*, and not as ∼1/*r*.

## Supporting Information Available

Additional data, in graphical form, that complements the numerical results presented in the main text (PDF)
