# Supplementary material for: Plateaus in the Potentials of Density-Functional Theory: Analytical Derivation and Useful Approximations
Source: J Chem Theory Comput. 2025 Mar 27;21(7):3476–92. doi: 10.1021/acs.jctc.4c01771 (PMC11983711; doi:10.1021/acs.jctc.4c01771)
Supplement: Supplementary file 1 — ct4c01771_si_001.pdf [file ct4c01771_si_001.pdf]

Supporting Information for: Plateaus in the potentials of density-functional theory: analytical derivation and useful approximations

Nathan E. Rahat[1] and Eli Kraisler[1,*]

[1] *Fritz Haber Research Center for Molecular Dynamics and Institute of Chemistry, The Hebrew University of Jerusalem, 9091401 Jerusalem, Israel*

(Dated: March 11, 2025)

I. THE FUNCTION Θ AND ITS APPROXIMATIONS

Figure S1 shows the difference $\Theta(r;\alpha) - \Omega(r;\alpha)$ for the Li atom with $2+\alpha$ electrons, along with the function $K(\alpha)\Theta(r;\alpha)(1-\Theta(r;\alpha))$. In the latter function, the factor $K(\alpha)$ is fitted in such a manner that the peak height (but not necessarily the peak position) of the two expressions is the same. We find a reasonably close correspondence between the two expressions, although as $\alpha \to 0^+$, and the peaks are travelling away from the center of the system, the $K\Theta(1-\Theta)$ function is slightly, but systematically, behind. Figure S1 demonstrates that $\Theta - \Omega$ is definitely an intermediate term, but also that its magnitude is logarithmically growing as $\alpha \to 0^+$.

Figure S2 compares $\Theta(r;\alpha) - \Omega(r;\alpha)$ to the relaxation term $\left(|\varphi_{1s}(r;\alpha)|^2 - |\varphi_{1s}(r;0)|^2\right) / \left(\frac{1}{2}n(r;\alpha)\right)$, numerically verifying Eqs. (32) and (33) of the main text. Figure S2 shows that the graphs for the two expressions overlap for all values of α.

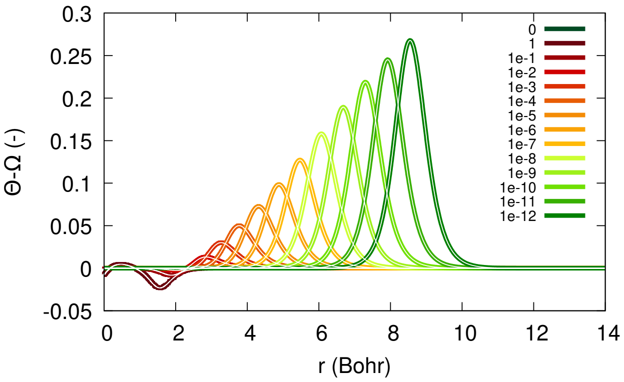

FIG. S2. Colored: The difference $\Theta(r;\alpha) - \Omega(r;\alpha)$, versus r, for Li with $2+\alpha$ electrons, for various values of α (see Legend). White: The function $\left(|\varphi_{1s}(r;\alpha)|^2 - |\varphi_{1s}(r;0)|^2\right) / \left(\frac{1}{2}n(r;\alpha)\right)$ (cf. Eqs. (32) and (33) of the main text). Full overlap with the colored graphs is observed.

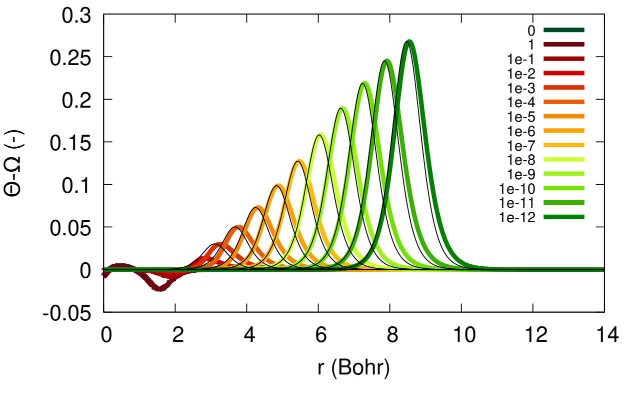

FIG. S1. Colored: The difference $\Theta(r;\alpha) - \Omega(r;\alpha)$, versus r, for Li with $2+\alpha$ electrons, for various values of α (see Legend). Black: The function $K(\alpha)\Theta(r;\alpha)(1-\Theta(r;\alpha))$, where $K(\alpha)$ is fitted to reproduce the peak height of $\Theta - \Omega$, for each α

* Author to whom correspondence should be addressed: eli.kraisler@mail.huji.ac.il

II. THE KSP PLATEAU FUNCTION

Figure S3 depicts, for various values of α, the expression that appears in the second line of Eq. (19) of the main text, which equals $P_{\mathrm{KSP}}(r;\alpha) - E_g$, where $E_g = \lim_{\alpha\to 0^+} \varepsilon_{\mathrm{ho}}(\alpha) - \varepsilon_{\mathrm{ho}}(0^-)$ is the fundamental gap of the system. We clearly see that for all α's the graphs approach the same limit at $r \to \infty$.

To computationally obtain this limit, it is advantageous to plot $P_{\mathrm{KSP}}(r;\alpha) - E_g$ versus $1/r$ (see Fig. S4). Then, for low values of $1/r$ (i.e., high values of r) we expect a linear dependence. Recalling that the numerical inversion procedure was required for $r \in [0.005, 29]$ Bohr, we are not surprised of the numerical noise that appears for $1/r < 0.035$. Furthermore, we find a linear behavior for all the curves in the interval $1/r = 0.055 - 0.095$ Bohr^{-1}. Fitting a straight line such that it passes through the curve points at 0.06 and 0.08 Bohr^{-1}, we find the fundamental gap to equal $E_g = -2.6895$ Hartree, in close correspondence to the eigenvalues obtained for Li.

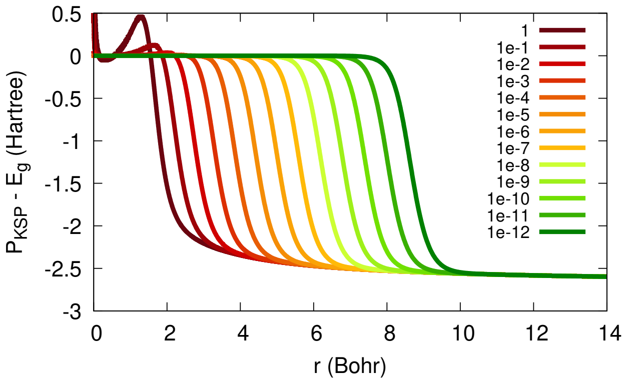

FIG. S3. The expression in the second line of Eq. (19) of the main text, versus r, for various values of α (see Legend)

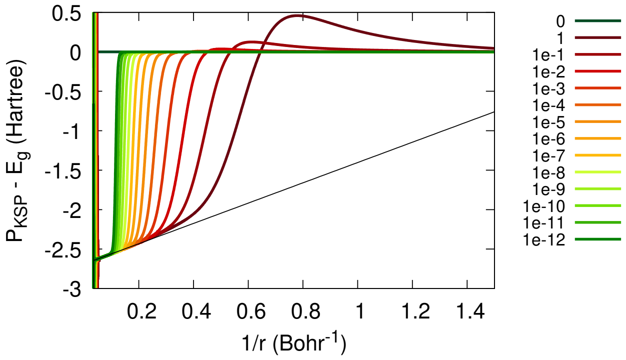

FIG. S4. The expression in the second line of Eq. (19) of the main text, versus $1/r$, for various values of α (see Legend)

III. THE PAULI PLATEAU FUNCTION

Figures S5, S6, S7, S8, S9, S10, S11 and S12 depict the nine terms of the Pauli plateau function, as it appears in Eq. (43) of the main text. For reasons explained in the main text, the third and the ninth terms are presented together. The graphical presentation of the various terms of the Pauli potential supports the analysis given in the main text, and hence the strategy we pursued to derive the simple, approximate form of the Pauli plateau, Eq. (50), appears justified.

Furthermore, Fig. S13 presents the sum of all the nine terms of the Pauli plateau (in color) versus the exact Pauli plateaus obtained from potential differences (in white; full overlap). With this figure, we verify the analytical derivation of Eq. (43).

Finally, Fig. S14 presents the sum of the third and ninth terms of the Pauli plateau of Eq. (43) (in color) versus its approximation, Eq. (49) (in black), to show the satisfactory accuracy of the suggested approximation.

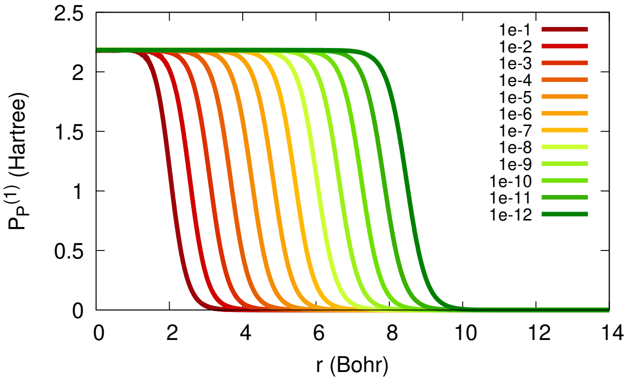

FIG. S5. The 1st term in Eq. (43) of the main text, for the Li atom with $2+\alpha$ electrons, for various values of α (see Legend)

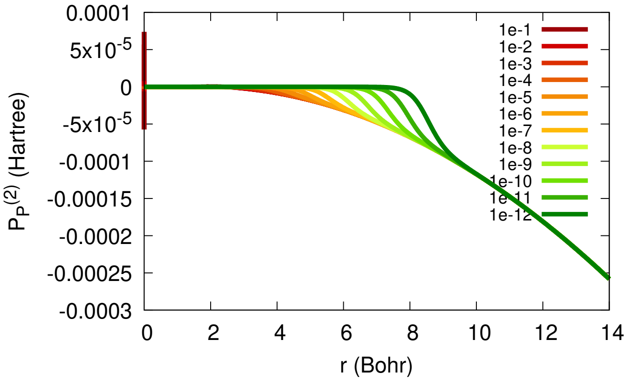

FIG. S6. The 2nd term in Eq. (43) of the main text, for the Li atom with $2+\alpha$ electrons, for various values of α (see Legend)

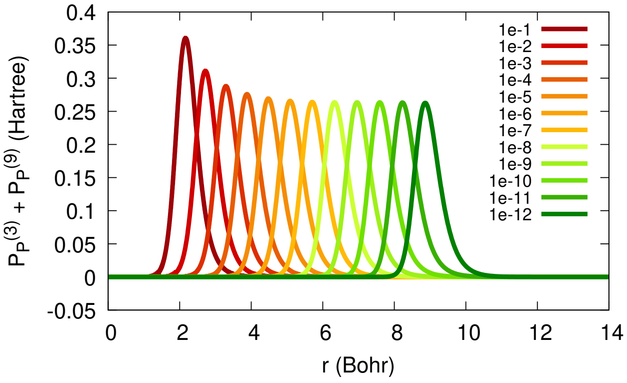

FIG. S7. Sum of the 3rd and the 9th terms in Eq. (43) of the main text, for the Li atom with $2 + \alpha$ electrons, for various values of α (see Legend)

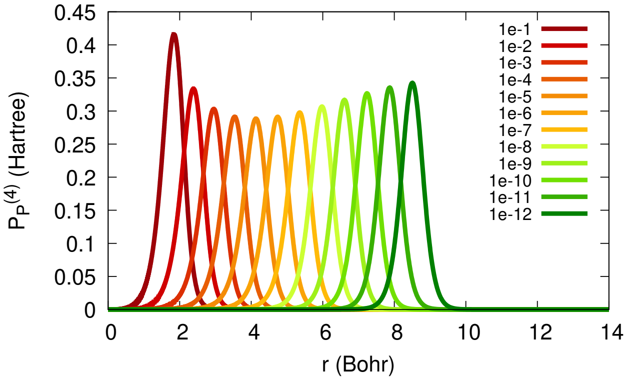

FIG. S8. The 4th term in Eq. (43) of the main text, for the Li atom with $2+\alpha$ electrons, for various values of α (see Legend)

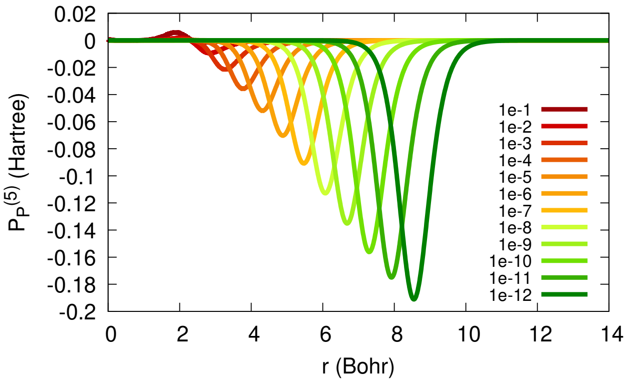

FIG. S9. The 5th term in Eq. (43) of the main text, for the Li atom with $2+\alpha$ electrons, for various values of α (see Legend)

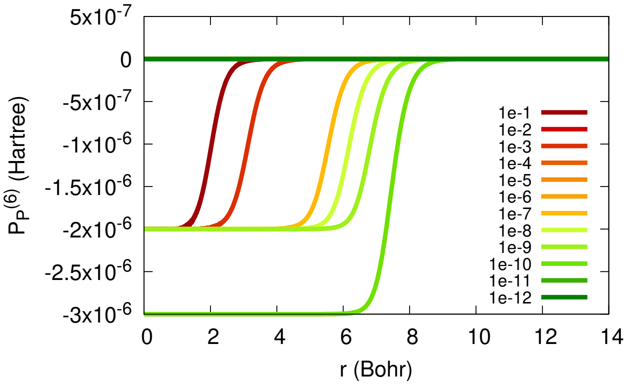

FIG. S10. The 6th term in Eq. (43) of the main text, for the Li atom with $2 + \alpha$ electrons, for various values of α (see Legend)

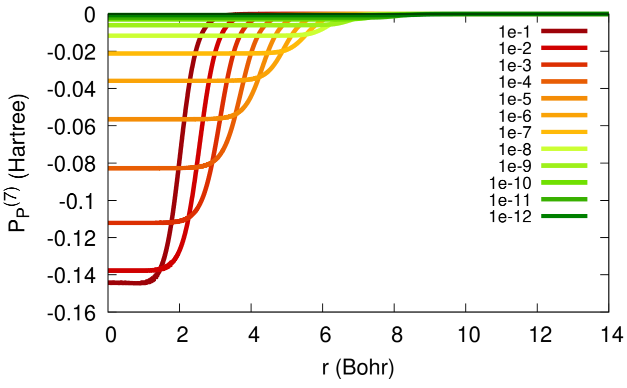

FIG. S11. The 7th term in Eq. (43) of the main text, for the Li atom with $2 + \alpha$ electrons, for various values of α (see Legend)

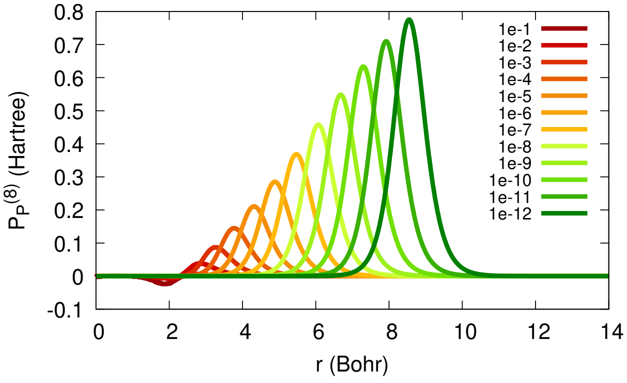

FIG. S12. The 8th term in Eq. (43) of the main text, for the Li atom with $2 + \alpha$ electrons, for various values of α (see Legend)

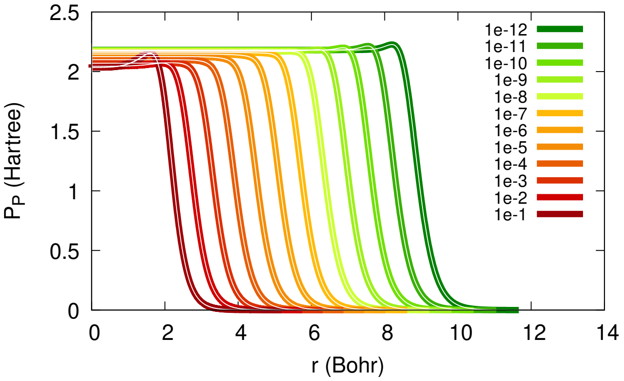

FIG. S13. Sum of all the nine terms of Eq. (43) of the main text (colored; see Legend), versus the exact Pauli plateau from potential differences (white), for the Li atom with $2+\alpha$ electrons, for various values of α. Full overlap is observed.

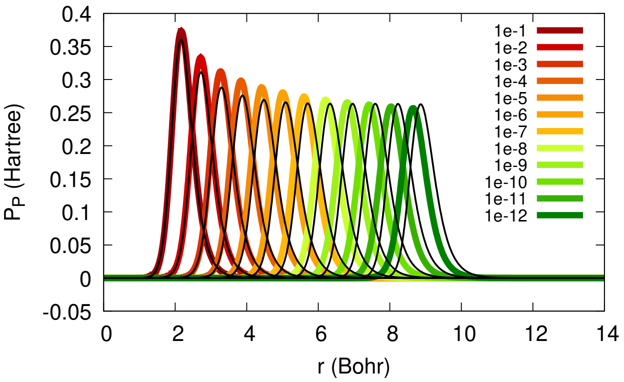

FIG. S14. Sum of the 3$^{\rm rd}$ and the 9$^{\rm th}$ terms in Eq. (43) of the main text, versus the approximation to this sum as in Eq. (49) of the main text, for the Li atom with $2+\alpha$ electrons, for various values of α (see Legend)

IV. THE FUNCTIONS Θ_σ AND Ω_σ FOR LSDA CALCULATIONS

Figure S15 depicts the function $\Theta_\uparrow(r;\alpha)$ obtained for the Li atom with $2+\alpha$ electrons, from densities that were obtained with LSDA calculations. In black, the exact Θ-function, obtained from FCI densities, is drawn for comparison. Although different both in slope and in position from the system center for a given α, the LSDA Θ-function reproduces the main features of the exact one: it approaches 0 near the origin and 1 at infinity, and it drifts from the center of the system at a logarithmic pace, as $\alpha \to 0^+$.

Figure S16 shows both $\Theta_\uparrow(r;\alpha)$ and $\Omega_\uparrow(r;\alpha)$ for Li with LSDA. As in the exact case, these functions are very close to each other, and their difference is an intermediate-region term, as can be seen in Fig. S17. Also for LSDA, the difference $\Theta_\uparrow(r;\alpha) - \Omega_\uparrow(r;\alpha)$ can be closely approximated by the function $K(\alpha)\Theta_\uparrow(r;\alpha)(1 - \Theta_\uparrow(r;\alpha))$, where $K(\alpha)$ is fitted to reproduce the peak of $\Theta_\uparrow(r;\alpha) - \Omega_\uparrow(r;\alpha)$, for each α. Remarkably, the values of $K(\alpha)$ for LSDA are very similar to those of the FCI case.

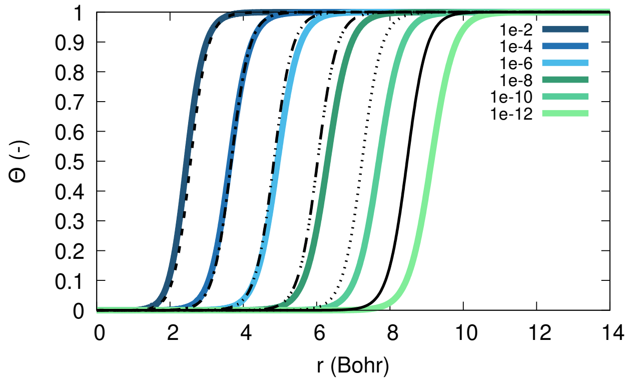

FIG. S15. Colored: The function $\Theta_\uparrow(r;\alpha)$, obtained from LSDA calculations for the Li atom with $2+\alpha$ electrons (see Legend). Black: The exact function $\Theta(r;\alpha)$, obtained from FCI calculations (presented in the main text), for $\alpha = 10^{-2}$ (dashed), $\alpha = 10^{-4}$ (dash-dot), $\alpha = 10^{-6}$ (dash-dot-dot), $\alpha = 10^{-8}$ (dash-3 dots), $\alpha = 10^{-10}$ (dot), $\alpha = 10^{-12}$ (solid).

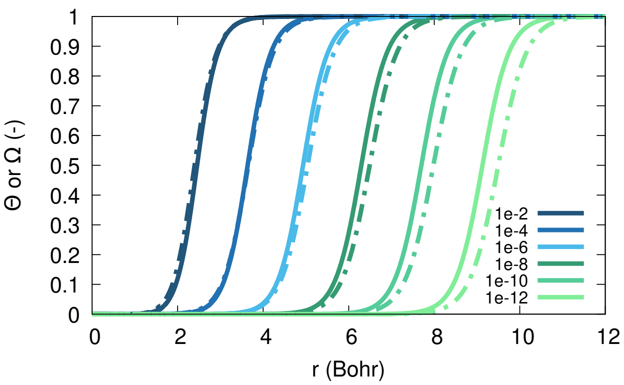

FIG. S16. Solid lines: The function $\Theta_\uparrow(r;\alpha)$, obtained from LSDA calculations for the Li atom with $2+\alpha$ electrons (see Legend). Dashed lines: same, for $\Omega_\uparrow(r;\alpha)$

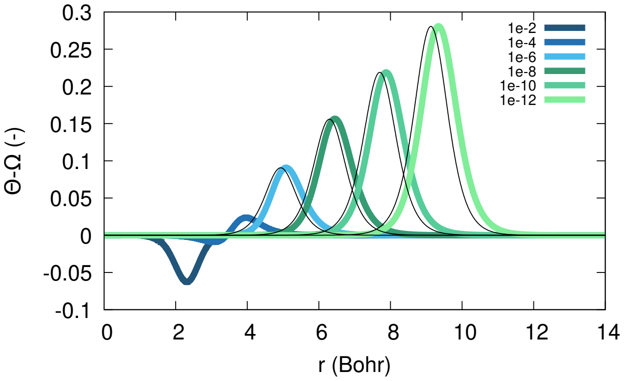

FIG. S17. Colored: The difference $\Theta_\uparrow(r;\alpha) - \Omega_\uparrow(r;\alpha)$, versus r, for Li with $2+\alpha$ electrons calculated with the LSDA, for various values of α (see Legend). Black: The function $K(\alpha)\Theta_\uparrow(r;\alpha)(1 - \Theta_\uparrow(r;\alpha))$, where $K(\alpha)$ is fitted to reproduce the peak of $\Theta_\uparrow(r;\alpha) - \Omega_\uparrow(r;\alpha)$, for each α
